# Multi-locus phylogenetic analyses uncover species boundaries and reveal the occurrence of two new entomopathogenic nematode species, *Heterorhabditis ruandica* n. sp. and *Heterorhabditis zacatecana* n. sp.

**DOI:** 10.21307/jofnem-2021-089

**Published:** 2021-11-11

**Authors:** Ricardo A.R. Machado, Aashaq Hussain Bhat, Joaquín Abolafia, Arthur Muller, Pamela Bruno, Patrick Fallet, Carla C.M. Arce, Ted C.J. Turlings, Julio S. Bernal, Joelle Kajuga, Bancy Waweru, Stefan Toepfer

**Affiliations:** 1 Experimental Biology Research Group. Institute of Biology. Faculty of Sciences. University of Neuchâtel. Rue Emile-Argand 11, 2000 Neuchâtel, Switzerland; 2 Department of Zoology, Government Degree College. Billawar-184204, Kathua, Jammu, Jammu and Kashmir, India; 3 Departamento de Biología Animal, Biología Vegetal y Ecología, Universidad de Jaén, Campus ‘Las Lagunillas’ s/n, Edificio B3, 23071 Jaén, Spain; 4 Laboratory of Fundamental and Applied Research in Chemical Ecology, Institute of Biology. Faculty of Sciences, University of Neuchâtel, 2000 Neuchâtel, Switzerland; 5 Department of Entomology, Texas A&M University, College Station, TX; 6 Department of Crop Innovations & Technology Transfer. Rwanda Agriculture and Animal Resources Development Board, 5016 Kigali-Rwanda; 7 CABI Switzerland, 2800 Delémont, Switzerland

**Keywords:** Biocontrol agents, Dichotomous key, Entomopathogenic nematodes, Nematode morphology, Photorhabdus, Phylogenetics, Species description, *Taxonomy*

## Abstract

Species of the nematode genus *Heterorhabditis* are important biological control agents against agricultural pests. The taxonomy of this group is still unclear as it currently relies on phylogenetic reconstructions based on a few genetic markers with little resolutive power, specially of closely related species. To fill this knowledge gap, we sequenced several phylogenetically relevant genetic loci and used them to reconstruct phylogenetic trees, to calculate sequence similarity scores, and to determine signatures of species- and population-specific genetic polymorphism. In addition, we revisited the current literature related to the description, synonymisation, and declaration as *species inquirendae* of *Heterorhabditis* species to compile taxonomically relevant morphological and morphometric characters, characterized new nematode isolates at the morphological and morphometrical level, and conducted self-crossing and cross-hybridization experiments. The results of this study show that the sequences of the mitochondrial cytochrome C oxidase subunit I (COI) gene provide better phylogenetic resolutive power than the sequences of nuclear rRNA genes and that this gene marker can phylogenetically resolve closely related species and even populations of the same species with high precision. Using this gene marker, we found two new species, *Heterorhabditis ruandica* n. sp. and *Heterorhabditis zacatecana* n. sp. A detailed characterization of these species at the morphological and morphometric levels and nematode reproduction assays revealed that the threshold for species delimitation in this genus, using *COI* sequences, is 97% to 98%. Our study illustrates the importance of rigorous morphological and morphometric characterization and multi-locus sequencing for the description of new species within the genus *Heterorhabditis*, serves to clarify the phylogenetic relationships of this important group of biological control agents, and can inform future species descriptions to advance our efforts towards developing more tools for sustainable and environmentally friendly agriculture.

Nematodes of the genus *Heterorhabditis* Poinar, 1976 are soil-dwelling organisms that parasitize and kill certain small arthropods, mainly insects (Kaya and Gaugler, 1993). Their lifestyle is particularly interesting as they establish an obligated, mutualistic symbiosis with entomopathogenic bacteria of the genus *Photorhabdus* (Clarke, 2020; Machado et al., 2018). Nematodes colonize their prey, and upon sensing unknown chemical cues, they release their symbiotic bacterial partners inside the bodies of the infected organisms (Ciche et al., 2008; Dillman et al., 2012). The bacteria establish, multiply and produce an arsenal of immunosuppressors, lytic enzymes, and toxins that kill the infected organism and pre-digest its tissues, which serve as food for the bacteria and the nematodes (Shankhu et al., 2020; Tobias et al., 2016; Vlisidou et al., 2019). The nematodes grow, reproduce, and, upon resource depletion, reestablish symbiosis with *Photorhabdus* bacteria, and abandon the consumed cadavers in search for new prey (Somvanshi et al., 2012). Given this peculiar lifestyle, this deadly symbiotic pair is commonly used as a biocontrol agent in agricultural settings (Kajuga et al., 2018; Paddock et al., 2021; Toepfer and Zellner, 2017; Zhang et al., 2019). In addition, given the enormous biosynthetic capacity of *Photorhabdus* bacteria, they are of great medical, agricultural, and biotechnological importance (Blackburn et al., 1998; Bode, 2009; Hill et al., 2020; Joyce and Clarke, 2003; Lacey and Georgis, 2012; Machado et al., 2018; Machado et al., 2020; Tobias et al., 2018).

The number of described species of the genus *Heterorhabditis* is steadily growing, mainly boosted by recent advances in genomics. Up to now, the genus includes between 16 and 21 valid species, several synonymized species and some *species inquirendae* (Boemare et al., 1993; Hunt and Nguyen, 2016; Maneesakorn et al., 2011; Sudhaus, 2011; Tóth and Lakatos, 2008). Given the discrepancy in the number of recognized valid species and the increasing number of synonymized species, a throughout revision of the current literature related to the description, synonymisation, and declaration as *species inquirendae* of *Heterorhabditis* species may help to determine the actual number of valid species in this genus. As some species were described prior to the discovery of modern molecular techniques, and therefore the sequences of phylogenetically relevant gene markers are not available, morphological characters play an important role in this context (Andaló et al., 2006; Edgington et al., 2011; Hunt and Nguyen, 2016; Liu and Berry, 1996; Li et al., 2012; Malan et al., 2008; Malan et al., 2014; Nguyen et al., 2004, 2006, 2008; Pereira, 1937; Phan et al., 2003; Poinar and Veremchuk, 1970; Poinar, 1971, 1976; Poinar et al., 1987, 1992; Poinar, 1990; Stock et al., 2002).

Ribosomal RNA (rRNA) gene sequences such as ITS sequences and the sequences of the D2–D3 expansion segments of the 28S rRNA are traditionally used for identification purposes and for novel taxonomic status descriptions of the species of the genus *Heterorhabditis* (Adams et al., 1998; Campos‐Herrera et al., 2011; Li et al., 2012; Malan et al., 2008; Nguyen et al., 2008; Rana et al., 2020; Spiridonov and Subbotin, 2016). As a recently evolved group, marginal variations in the rRNA gene sequences are expected in this genus, which limits the use of these genetic markers for taxonomic purposes, especially of closely related species (Blaxter et al., 1998; Blouin, 2002; Haag et al., 2018). In addition, the use of sequences containing several ambiguous nucleotides, potentially arisen from sequencing errors and/or poor quality-control, leads to erroneous taxonomic affiliations, as it is exemplified by the relatively high number of synonym species in the genus *Heterorhabditis* (Dhakal et al., 2020; Hunt and Nguyen, 2016). The use of mitochondrial DNA such as *COI* sequences, the gold standard taxonomic marker for species delimitation in the Kingdom Animalia, may help to overcome the taxonomic limitations of rRNA gene sequences. However, this taxonomic marker has been used only sporadically for identification purposes, barely used for taxonomy, and never used to describe new *Heterorhabditis* species (Chaubey et al., 2016; Hebert et al., 2003; Joyce et al., 1994a; Kuwata et al., 2007). As a consequence, the availability of *COI* sequences for this genus remained very limited for several years, limiting our understanding of the phylogenetic relationships of this genus (Chaubey et al., 2016; Dhakal et al., 2020; Kuwata et al., 2007).

To improve our understanding on the phylogenetic relationships of *Heterorhabditis* nematodes, to determine the most suitable genetic markers for the rapid and reliable identification of the species of this genus, specially of closely related species, and to determine species boundaries in this genus, we generated nucleotide sequences of several phylogenetically relevant gene markers and used them to reconstruct phylogenetic trees, to calculate sequence similarity scores, and to determine signatures of species- and population-specific genetic polymorphism. To improve our understanding on the taxonomic relationships of *Heterorhabditis* nematodes, we revisited the current literature related to the description, synonymisation, and declaration as *species inquirendae* of *Heterorhabditis* species to compile taxonomically relevant morphological and morphometric characters, characterized new nematode isolates at the morphological and morphometrical level, and conducted self-crossing and cross-hybridization experiments. Our study illustrates the importance of multi-locus sequencing for the characterization of new species within the genus *Heterorhabditis*, serves to clarify the phylogenetic relationships of these important biological control agents, and can inform future species descriptions to advance our efforts towards developing more tools for sustainable and environmentally friendly agriculture.

## Materials and methods

### Nematode origin

*Heterorhabditis* nematodes used in this study were collected by us during different nematode collection campaigns carried out in Rwanda, Mexico, and India, or were collected by different collaborators at different locations around the world (Table S1) (Bai et al., 2013; Bhat et al., 2021b; Bruno et al., 2020; Carrera, 2015; Fallet et al., 2020; Mukuka et al., 2010; Rana et al., 2020; Yan et al., 2016).

### Nematode morphological and morphometrical characterization, light, and scanning electron microscopy

One representative nematode isolate of each new species, MEX-39 and Rw14_N-C4a, was selected for detailed morphological and morphometrical characterization. First- and second-generation adult nematodes were obtained by dissecting infected *G. mellonella* larvae in Ringer’s solution. Infective juveniles (IJs) were collected after their emergence from *G. mellonella* larvae in White traps (White, 1927). Nematodes were killed with water at 60°C, then fixed in triethanolamine formalin (7  ml formalin, 2  ml triethanolamine, 91 ml ddH_2_O), then dehydrated by the Seinhorst’s method, and finally transferred to glycerine (Bhat et al., 2019b; Courtney et al., 1955; Seinhorst, 1959, 1962). Nematodes were mounted in small drops of glycerine on permanent glass slides with extra layers of paraffin wax to prevent the flattening of the nematodes (Bhat et al., 2021a). Morphological measurements were taken using the Nikon DS-L1 software built in a phase contrast microscope (Nikon Eclipse 50i). Between 20 and 25 specimens at each developmental stage were measured. Light microscopy photographs were taken using a Nikon Eclipse 80i microscope (Nikon, Tokyo, Japan) equipped with differential interference contrast optics (DIC) and a Nikon Digital Sight DS–U1 camera. For the scanning electron microscopy (SEM), nematode specimens preserved in glycerine were processed as described by Abolafia (2015). For this, the nematodes were re–hydrated in distilled water, dehydrated in ethanol-acetone, critical-point dried with liquid carbon dioxide, mounted on SEM stubs with copper tape and coated with gold in a sputter coater. Specimens were observed and microphotographs were captured using a Zeiss Merlin microscope (5 kV) (Zeiss, Oberkochen, Germany). All micrographs were processed using Adobe® Photoshop® CS. The obtained morphometrical characters were compared with those published in previous studies describing all the species of the genus, independently of their current status (valid, *species inquirendae*, synonym, etc) (Abd-Elgawad and Ameen, 2005; Agüera de Doucet and Doucet, 1986; Andaló et al., 2006; Bhat et al., 2019a; Bhat et al., 2021b; Edgington et al., 2011; Gardner et al., 1994; Hunt and Nguyen, 2016; Kajol et al., 2020; Kakulia and Mikaia, 1997; Khan et al., 1976; Liu, 1994; Liu and Berry, 1996; Li et al., 2012; Malan et al., 2008; Malan et al., 2014; Maneesakorn et al., 2015; Nguyen et al., 2004; Nguyen et al., 2006; Nguyen et al., 2008; Pereira, 1937; Phan et al., 2003; Plichta et al., 2009; Poinar and Veremchuk, 1970; Poinar, 1976; Poinar et al., 1987; Poinar et al., 1992; Rana et al., 2020; Sagun et al., 2015; Shahina et al., 2017; Shamseldean et al., 1996; Stock, 1993; Stock et al., 1996; Stock, 1997; Stock et al., 2002; Stock et al., 2009; Turco, 1970; Vanlalhlimpuia et al., 2018; Wouts, 1979).

### Self-crossing and cross-hybridization experiments

Self-crossing and cross-hybridization experiments were carried out on lipid agar plates as described by Dix et al. (1992). *Heterorhabditis ruandica* n. sp. Rw14_N-C4a and *H. zacatecana* n. sp. MEX-39 were self-crossed, hybridized with each other and with *H. bacteriophora* CH21 (Rana et al., 2020). For this, one second–generation male and one second–generation virgin female were placed on lipid agar plates (35 mm diam.) and incubated at 27°C. Ten independent plates per crossing type were set. Progeny production was observed daily for a period of five consecutive days. Experiments were repeated three times under the same conditions.

### Nematode molecular characterization and phylogenetic relationships

Genomic DNA from about 10 to 20 thousand nematodes was extracted using the genomic DNA isolation kit following manufacturer’s instructions (Norgen Biotek Corp., Thorold, Ontario, Canada). The following genes/genomic regions were amplified by polymerase chain reaction (PCR): the D2–D3 expansion segments of the 28S rRNA, the internal transcribed spacer (ITS) region of the rRNA, the cytochrome c oxidase I (*COI*), the thin filament (F-actin)-associated protein (*unc-87*), and the calmodulin 1 (*cmd-1*). Primers used were selected based on previous publications (Dhakal et al., 2020; Joyce et al., 1994b; Regeai et al., 2009; Subbotin et al., 2006) (Table S2). PCR reactions consisted of 1 µL of genomic DNA, 12.5 µL of EmeraldAmp GT PCR Master Mix (Takara Bio, Shiga, Japan), 0.5 µL of both forward and reverse primers at 10 mM and 10.5 µL of dH_2_O. The PCR reaction was performed using a thermocycler (Mastercycler nexus gradient, Eppendorf, Germany) with the following settings: (i) for ITS and D2–D3, 1 cycle of 1 min at 98°C followed by 35 cycles of 10 sec at 98°C, 30 sec at 50°C, 1 min 30 sec at 72°C, and by a single final elongation step at 72°C for 10 min; (ii) for *cmd-1* and *unc-87*, 1 cycle of 1 min at 98°C followed by 40 cycles of 10 sec at 98°C, 30 sec at 50°C, 30 sec at 72°C, and by a single final elongation step at 72°C for 10 min; (iii) for *COI*, 1 cycle of 1 min at 98°C followed by 40 cycles of 10 sec at 98°C, 30 sec at 40°C, 30 sec at 72°C, and by a single final elongation step at 72°C for 10 min. PCR was followed by electrophoresis (45 min, 100 V) of 5 μl of PCR products in a 1% TBA (Tris–boric acid–EDTA) buffered agarose gel stained with SYBR Safe DNA Gel Stain (Invitrogen, Carlsbad, California, USA). PCR products were purified using the FastGene Gel/PCR extraction kit (Nippon Genetics Co., Japan) and sequenced using reverse and forward primers by Sanger sequencing (Microsynth AG, Balgach, Switzerland). Obtained sequences were manually curated and trimmed and deposited in the NCBI under the accession numbers given in Table S3. Sequences of the following nematode strains were obtained in this study: *Heterorhabditis ruandica* n. sp. (isolates Rw18_M-Hr1a, Rw18_M-Hr1b, and Rw14_N-C4a), *H. zacatecana* n. sp. (isolates MEX-39, MEX-40, and MEX-41), *H. bacteriophora* (isolates DE2, DE6, PT1, IT6, EN01, and TT01); *H. georgiana* Hbb, *H. beicherriana* H06, *H. indica* CH7, and *H. atacamensis* MEX-20. To complete this data set and to obtain genomic sequences of nematodes that belong to all the validly described species of the genus *Heterorhabditis*, we searched the database of the National Center for Biotechnology Information (NCBI) by the Basic Local Alignment Search Tool (BLAST) using the accession numbers of the sequences obtained previously (Dhakal et al., 2020) (Table S3). Resulting sequences were used to reconstruct phylogenetic relationships by the Maximum Likelihood method based on the following nucleotide substitution models: Hasegawa–Kishino–Yano (HKY + I) (*cmd-1*), Tamura–Nei (TN93 + G + I) (*COI*), Kimura 2-parameter (K2 + G) (D2–D3 and ITS), and Tamura 3-parameter (T92) (*unc-87*). To select the best substitution model, best-fit nucleotide substitution model analyses were carried out in MEGA 7 (Hasegawa et al., 1985; Kimura, 1980; Kumar et al., 2016; Nei and Kumar, 2000). Sequences were aligned with MUSCLE (v3.8.31) (Edgar, 2004). The trees with the highest log likelihood are shown. The percentage of trees in which the associated taxa clustered together is shown next to the branches. Initial tree(s) for the heuristic search were obtained automatically by applying Neighbor-Join and BioNJ algorithms to a matrix of pairwise distances estimated using the Maximum Composite Likelihood (MCL) approach, and then selecting the topology with superior log likelihood value. In some cases, a discrete Gamma distribution (+G) was used to model evolutionary rate differences among sites and the rate variation model allowed for some sites to be evolutionarily (+I). The trees are drawn to scale, with branch lengths measured in the number of substitutions per site. Graphical representation and edition of the phylogenetic tree were performed with Interactive Tree of Life (v3.5.1) (Chevenet et al., 2006; Letunic and Bork, 2016).

### Symbiotic relationships

The *Photorhabdus* entomopathogenic bacteria associated with *H. ruandica* n. sp. Rw14_N-C4a and *H. zacatecana* n. sp. MEX-39 nematodes were isolated as described by Machado et al. (2019), (2021b). Briefly, *Galleria mellonella* larvae (Lepidoptera: Pyralidae) were exposed to 100 nematode infective juveniles. Three to four days later, insect cadavers were surface–sterilized and cut open with a blade. Bacteria-digested internal organs were spread onto LB agar plates and incubated at 28°C for 24 to 48 h. *Photorhabdus*-like colonies were sub-cultured until monocultures were obtained. A single primary form colony was then selected and used for further experiments. Bacteria primary forms were determined by examining colony characteristics and by examining pigments uptake on NBTA plates (LB agar plates supplemented with 25 mg l^–1^ bromothymol blue and 4 mg l^–1^ triphenyl-2,3,5-tetrazolium chloride). The strains were further sub-cultured and maintained on LB agar plates at 28°C. To establish their taxonomic identities, we reconstructed phylogenetic relationships based on whole genome sequences of the isolated bacteria and all the different species/subspecies of the genus *Photorhabdus* (Machado et al., 2021a, b). To obtain genomic sequences, genomic DNA was extracted and purified using the GenElute Bacterial Genomic DNA Kit (Sigma-Aldrich, Switzerland) following manufacturer’s instructions. The resulting DNA was used for library preparation using the TruSeq DNA PCR-Free LT Library Prep (FC-121-3003) kit. Indexed libraries were then pooled at equimolar concentrations and sequenced (2 × 150 bp) on an Illumina HiSeq 3000 instrument. Genomes were assembled using the Bactopia pipeline (Petit and Read, 2020). Briefly, the raw Illumina reads were quality trimmed using Trimmomatic 0.39 (Bolger et al., 2014). The resulting reads were assembled with SPAdes 3.14.1 (*k*-mer sizes of 31, 51, 71, 91, and 111 bp) (Bankevich et al., 2012). Scaffolds with a mean read-depth smaller than 20% of the median read-depth of the longer scaffolds (≥5,000 bp) as well as scaffolds that were shorter than 200 bp were removed. The final assemblies were polished using Pilon 1.22 (Walker et al., 2014). Genome sequences were deposited in the National Centre for Biotechnology Information. Accession numbers are listed in Table S4. Phylogenetic relationships were reconstructed based on the assembled genomes and the genome sequences of all validly published species of the genus (Machado et al., 2021a, b). For this, core genome alignments were created using Roary 3.6.2 (Page et al., 2015). Using this alignment, a maximum likelihood tree was constructed using Fasttree 2.1.10 based on the Jukes-Cantor + CAT nucleotide evolution model (Price et al., 2009, 2010). These analyses were carried out in Galaxy (Afgan et al., 2018). Whole genome sequence similarities were calculated by the digital DNA-DNA hybridization (dDDH) method using the recommended formula 2 of the genome-to-genome distance calculator (GGDC) web service of the Deutsche Sammlung von Mikroorganismen und Zellkulturen GmbH (DSMZ) (Auch et al., 2010a, 2010b; Meier-Kolthoff et al., 2013, 2014).

## Results and discussion

### Heterorhabditis ruandica n. sp.

**Figure 1:**
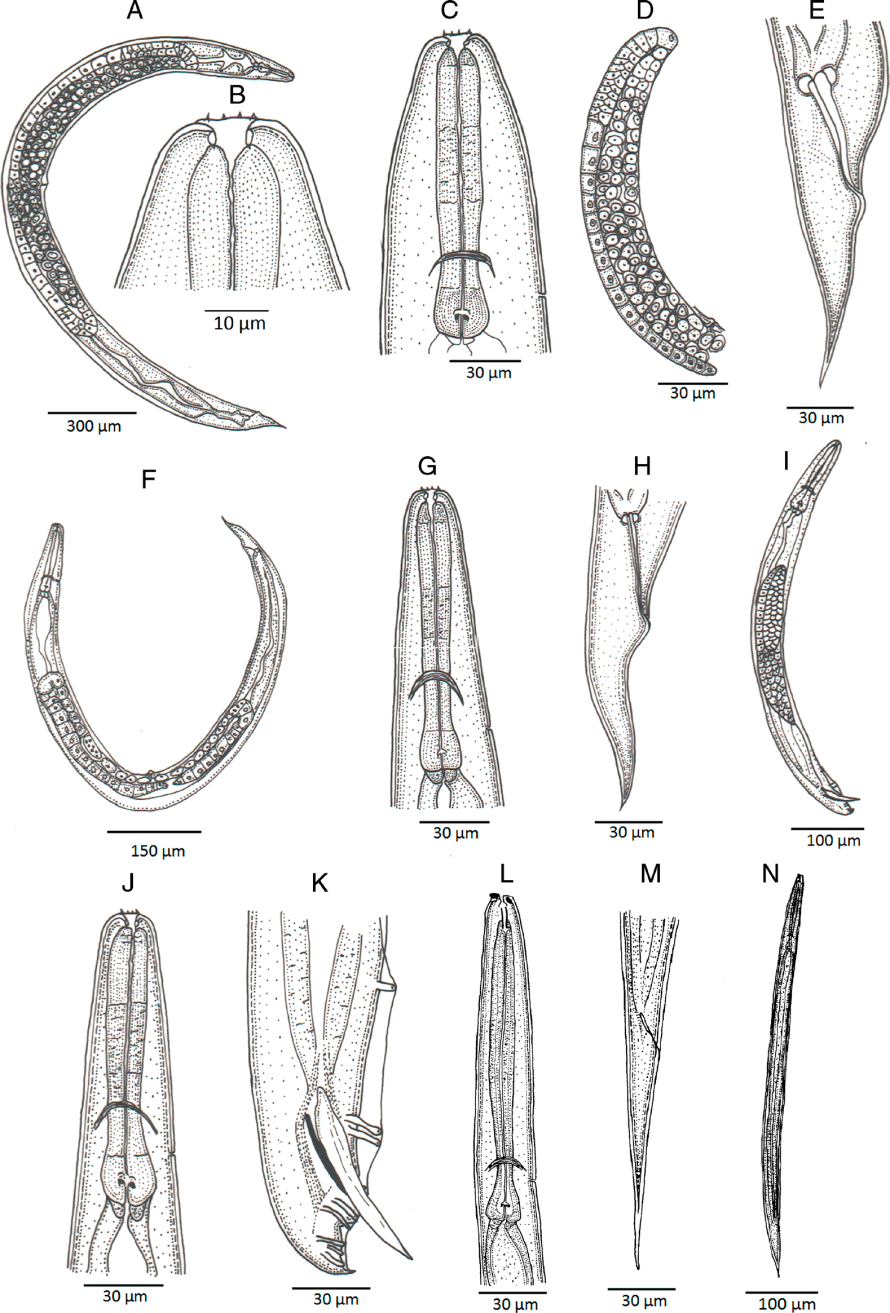
Line drawings of *Heterorhabditis ruandica* n. sp. (A) A hermaphroditic female. (B) Cephalic region of a hermaphroditic female. (C) Pharyngeal region of a hermaphroditic female. (D) Anterior part of the reproductive system of a hermaphroditic female. (E) Posterior end of a hermaphroditic female. (F) An amphimictic female. (G) Pharyngeal region of an amphimictic female. (H) Posterior end of an amphimictic female. (I) A male adult. (J) Pharyngeal region of a male adult. (K) Posterior region of a male adult. (L) Pharyngeal region of an infective juvenile. (M) Posterior end of an infective juvenile. (N) An infective juvenile.

**Figure 2:**
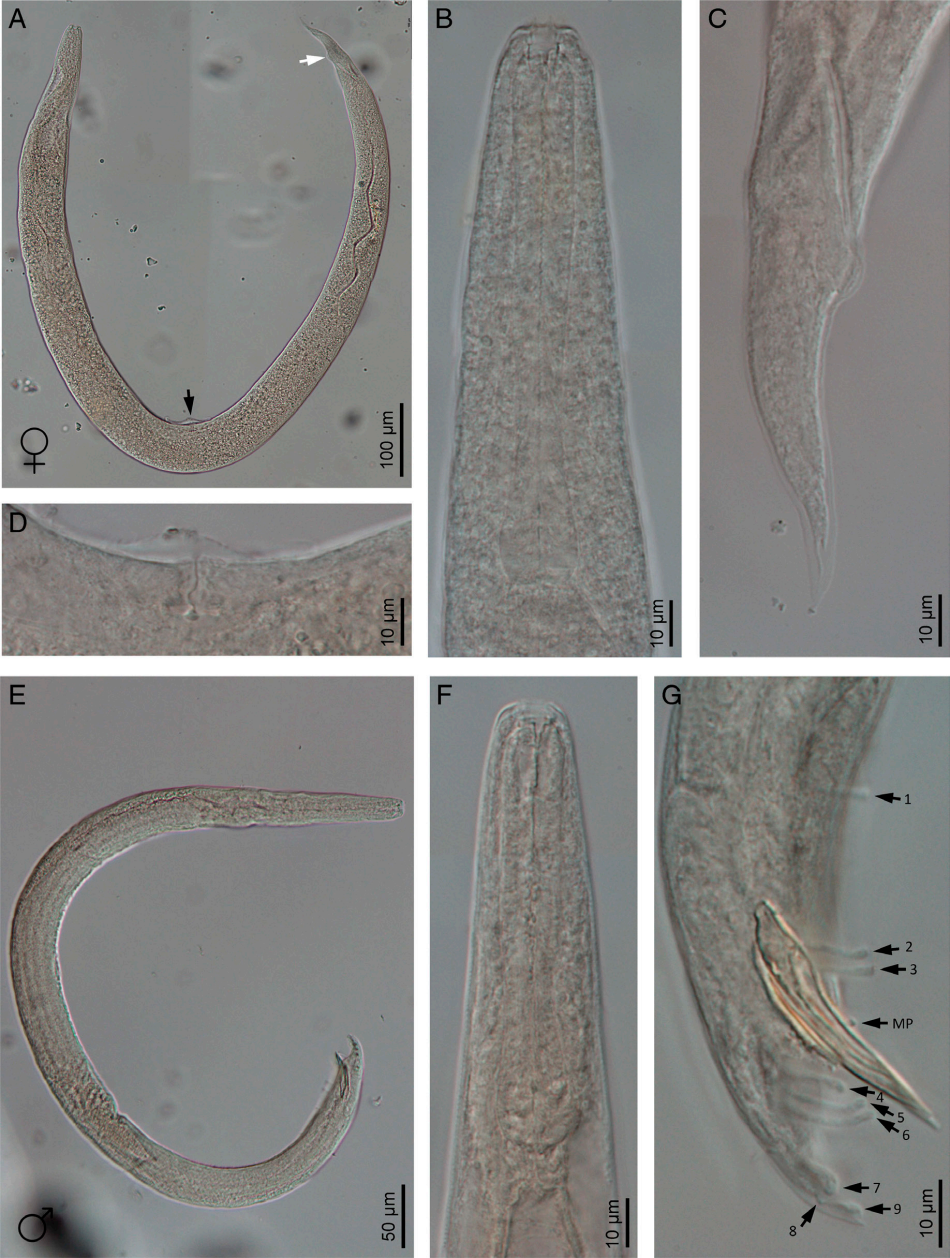
Light microscope micrographs of *Heterorhabditis ruandica* n. sp. (A) An amphimictic female (black arrow pointing at the position of the vulva, white arrow pointing at the anus). (B) Pharyngeal region of an amphimictic female. (C) Posterior end of an amphimictic female. (D) Vulva of an amphimictic female. (E) A male adult. (F) Pharyngeal region of a male adult. (G) Posterior end of a male adult (arrows pointing at the genital papillae).

**Figure 3:**
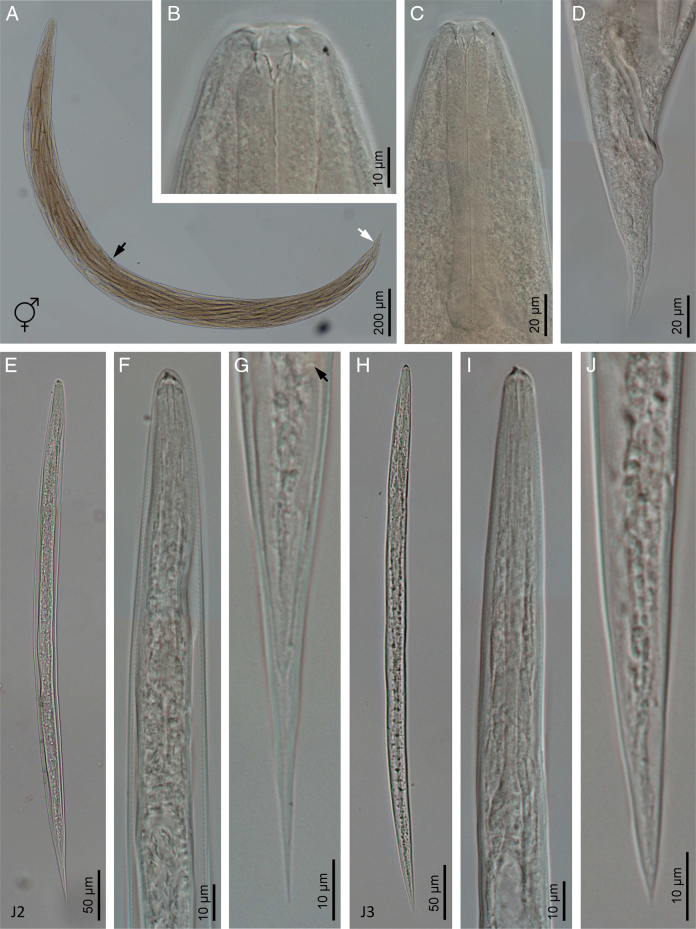
Light microscope micrographs of *Heterorhabditis ruandica* n. sp. (A) A hermaphroditic female. (B) Anterior end of a hermaphroditic female. (C) Pharyngeal region of a hermaphroditic female. (D) Posterior end of a hermaphroditic female. (E) A sheathed third stage juvenile (J2). (F) Pharyngeal region of a sheathed third stage juvenile (J2). (G) Posterior end of a sheathed third stage juvenile (J2) (arrow pointing the anus). (H) A non-sheathed third stage juvenile (J3). (I) Pharyngeal region of a non-sheathed third stage juvenile (J3). (J) Posterior end of a non-sheathed third stage juvenile (J3).

**Figure 4:**
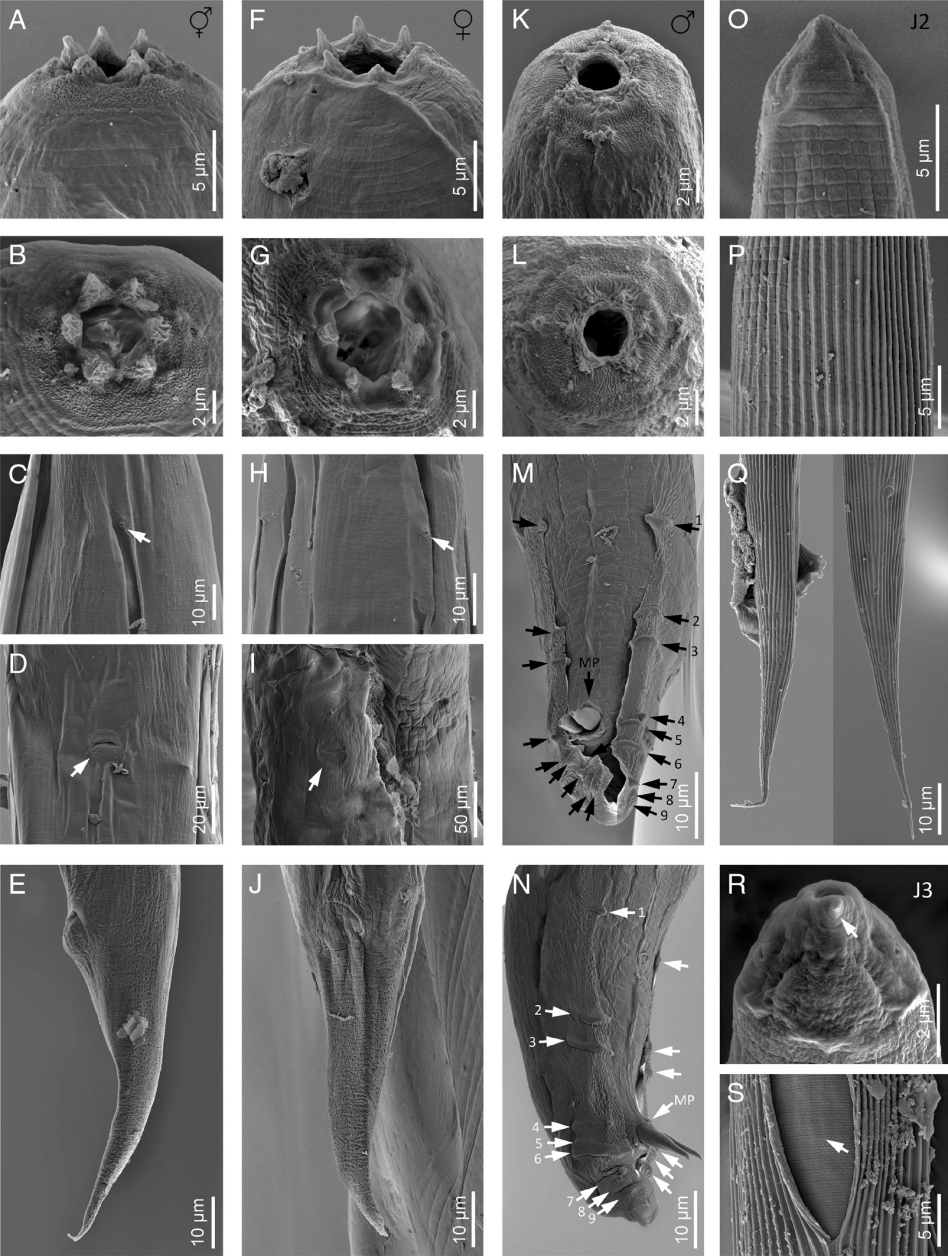
Scanning electron microscope (SEM) micrographs of *Heterorhabditis ruandica* n. sp. (A, B) Lip region in lateral and frontal views, respectively, of a hermaphroditic female. (C) Excretory pore of a hermaphroditic female (pointed by an arrow). (D) Vulva of a hermaphroditic female (pointed by an arrow). (E) Tail of a hermaphroditic female in lateral view. (F, G) Lip region of a female adult in lateral and frontal views, respectively. (H) Excretory pore (pointed by an arrow). (I) Vulva of a female adult (pointed by an arrow). (J) Tail of a female adult in ventral view. (K, L) Lip region of a male adult in sublateral and frontal views, respectively. (M, N) Posterior end of a male adult in ventral and lateral views, respectively (arrows pointing the genital papillae). (O) Lip region of a second-stage juvenile (J2) in lateral view. (P) Cuticle of a second-stage juvenile (J2). (Q) Tail of a second-stage juvenile (J2) in lateral and ventral views, respectively. (R) Lip region of a third-stage juvenile (J3) in ventral view (arrow pointing the frontal tooth). (S) Cuticle (arrow pointing the cuticle of a third-stage juvenile).

Figures 1–4, Tables 1 and 3–6.

**Table 1.** Morphometrics of infective juveniles and adult generations of *Heterorhabditis ruandica* n. sp.

	Male				
Characters	Holotype	Paratypes	Hermaphrodite (1^st^ Gen) paratypes	Female (2^nd^ Gen) paratypes	Infective juvenile paratypes
n	1	20	20	20	25
Body length (L)	760	769 ± 60 (652-863)	3295 ± 286 (2907-4123)	1366 ± 123 (1131-1608)	544 ± 29 (496-591)
a (L/BD)	20.3	17 ± 1.5 (15-21)	14.1 ± 1.1 (11.7-16.1)	18 ± 1.4 (15-20)	24 ± 3.0 (20-29)
b (L/NL)	7.8	8.1 ± 1.0 (5.8-9.7)	23 ± 1.8 (21-27)	11.4 ± 1.2 (9.0-13.6)	4.7 ± 0.4 (4.1-5.4)
c (L/T)	26.2	31 ± 3.6 (23-36)	42 ± 5.7 (34-51)	20 ± 2.2 (16-24)	8.2 ± 1.0 (7.6-8.6)
c’ (T/ABW)	1.1	1.4 ± 0.2 (0.6-1.7)	2.2 ± 0.3 (1.7-2.6)	2.8 ± 0.5 (1.9-3.6)	4.6 ± 0.8 (3.4-5.8)
V (VA/L × 100)	–	–	48 ± 2.5 (45-55)	48 ± 2.3 (41-51)	–
Max. Body Width (MBD)	37.5	44 ± 3.0 (40-51)	233 ± 17 (209-274)	77 ± 4.0 (68-83)	23 ± 2.7 (18-27)
Lip region width	6.5	7.2 ± 0.8 (5.7-8.4)	12.4 ± 0.8 (11.0-14.0)	10.3 ± 0.9 (8.8-12.2)	–
Stoma length	9.5	11.1 ± 1.6 (8.7-13.9)	14.9 ± 1.4 (13-18)	13.6 ± 1.8 (10.4-16.0)	13.8 ± 1.2 (12.1-16.0)
Bulb length (BL)	18.5	20 ± 1.8 (18-25)	35 ± 3.6 (29-42)	27 ± 2 (23-30)	13.8 ± 1.8 (11.0-19.0)
Pharynx length (PL)	95.2	84 ± 7.1 (74-107)	128 ± 6.3 (118-142)	107 ± 6.9 (91-120)	102 ± 7.0 (91-115)
Nerve ring – ant. end (NR)	68	63 ± 5.2 (56-74)	93 ± 7.5 (78-108)	81 ± 6.4 (69-97)	55 ± 3.6 (52-64)
Excretory pore– ant. end (EP)	84.3	81 ± 10.1 (61-109)	121 ± 11 (106-153)	111 ± 10.8 (92-129)	78 ± 3.4 (70-89)
Neck length (Stoma+Pharynx, NL)	98	96 ± 7.3 (84-117)	143 ± 6.3 (134-159)	120 ± 6.0 (107-132)	115 ± 7.3 (103-131)
Body width at neck base	36	34 ± 1.9 (30-37)	119 ± 8.9 (101-138)	58 ± 4.3 (50-66)	18 ± 3.0 (15-24)
Vagina length	–	–	28 ± 4.0 (20-38)	19.2 ± 2.9 (15-26)	–
Body width at vulva	–	–	240 ± 21 (199-278)	78 ± 3.8 (72-85)	–
Vulva – ant. end (VA)	–	–	1581 ± 151 (1369-1882)	655 ± 47 (572-706)	–
Vulva – post. End (PV)	–	–	1713 ± 178 (1453-2241)	710 ± 89 (559-949)	–
Rectum length	–	–	36 ± 4.6 (29-49)	30 ± 3.8 (24-35)	8.5 ± 1.9 (6.1-13.7)
Anal body diam. (ABD)	26.1	18 ± 2.4 (15-25)	37 ± 5.5 (29-51)	25 ± 4.5 (18-34)	12.4 ± 1.8 (9.2-16.0)
Tail with sheath length (T)	–	–	–	–	56 ± 4.9 (49-64)
Tail without sheath length	29	25 ± 3.2 (21-29)	80 ± 7.9 (63-98)	68 ± 6.5 (62-88)	30.4 ± 4.5 (22-39)
Spicule length (SL)	49	43 ± 4.1 (34-50)	–	–	–
Gubernaculum length (GL)	20.2	18 ± 1.5 (15-21)	–	–	–
Stoma length/lip region width	1.5	–	1.2 ± 0.2 (1.0-1.6)	1.3 ± 0.1 (1.1-1.6)	–
Nerve ring % (NR/NL × 100)	69.4	67 ± 4.4 (58-75)	65 ± 5.0 (56-78)	67 ± 3.9 (61-75)	–
Excretory pore % (EP/NL × 100)	86	85 ± 8.5 (61-97)	85 ± 8.3 (67-103)	92 ± 7.6 (74-104)	–
Rectum % (R/ABD × 100)	–	–	90 ± 10 (80-130)	128 ± 29 (90-181)	–
D % (EP /PL × 100)	88.5	96 ± 9.2 (69-111)	95 ± 9.3 (74-114)	104 ± 9.3 (82-118)	78 ± 7.6 (66-98)
E % (EP/T × 100)	290	325 ± 49 (232-413)	153 ± 24 (120-205)	164 ± 23 (111-203)	139 ± 13.4 (112-168)
SW % (SL/ABD × 100)	242	243 ± 47 (150-306)	–	–	–
GS % (GL/SL × 100)	41.2	42 ± 5.2 (35-57)	–	–	–
H % (H/T × 100	–	–	–	–	46 ± 4.0 (37–55)

**Table 2.** Morphometrics of infective juveniles and adult generations of *Heterorhabditis zacatecana* n. sp.

	Male				
Characters	Holotype	Paratypes	Hermaphrodite (1st Gen) paratypes	Female (2nd Gen) paratypes	Infective juvenile paratypes
*n*	1	20	22	22	25
Body length (L)	808.1	861 ± 29 (811-914)	5127 ± 494 (4408-6179)	2244 ± 203 (1954-2798)	539 ± 21 (493-578)
a (L/BD)	19.0	18 ± 1.6 (15-22)	16 ± 2.0 (13-20)	12.3 ± 1.2 (10.5-15.0)	22 ± 1.2 (19-24)
b (L/NL)	8.1	9.1 ± 1.1 (7.6-12)	26 ± 4.3 (20-34)	18 ± 1.8 (16-21)	5.0 ± 0.4 (4.4-5.9)
c (L/T)	28.9	34 ± 4.2 (26-43)	70 ± 10.4 (52-90)	39 ± 7.4 (31-63)	9.4 ± 0.6 (8.2-10.5)
c’ (T/ABW)	1.4	1.6 ± 0.3 (1.2-2.5)	1.6 ± 0.3 (1.2-2.4)	1.7 ± 0.2 (1.3-2.0)	5.3 ± 0.6 (4.3-6.7)
V (VA/L × 100)	–	–	48 ± 4.3 (36-57)	53 ± 4.2 (43-61)	–
Max. Body Width (MBD)	42.5	48 ± 3.6 (41-56)	319 ± 41 (235-385)	183 ± 23 (160-228)	24 ± 0.9 (23-27)
Lip region width	6.2	7.4 ± 0.7 (6.2-8.8)	11.7 ± 2.4 (9.2-19.2)	10.1 ± 1.0 (7.7-11.4)	4.0 ± 0.5 (3.2-5.2)
Stoma length	10	9.3 ± 1.0 (6.3-11)	19 ± 2.0 (14-23)	11.5 ± 1.7 (8.0-15.2)	13.5 ± 1.0 (12.0-15.3)
Bulb length (BL)	20.2	22 ± 2.4 (19-28)	40 ± 4.6 (28-49)	30 ± 2.6 (28-38)	20 ± 1.4 (17.1-23.0)
Pharynx length (PL)	95.2	86 ± 9.8 (57-100)	182 ± 23 (155-211)	113 ± 9.5 (101-133)	95 ± 7.2 (82-111)
Nerve ring – ant. end (NR)	65.4	66 ± 5.3 (60-78)	131 ± 22 (96-169)	83 ± 7.3 (71-96)	81 ± 6.3 (69-72)
Excretory pore– ant. end (EP)	96.2	93 ± 9.6 (77-109)	150 ± 24 (108-190)	113 ± 11 (100-133)	89 ± 6.8 (72-99)
Neck length (Stoma+Pharynx, NL)	99.3	96 ± 9.6 (71-108)	201 ± 21 (174-231)	124 ± 10 (112-148)	109 ± 6.9 (96-124)
Body width at neck base	34.5	36 ± 2.3 (31-40)	167 ± 13 (133-188)	95 ± 13.9 (74-121)	23 ± 1.3 (19-26)
Vagina length	–	–	31 ± 4.0 (24-36)	25 ± 6.4 (17-42)	–
Body width at vulva	–	–	331 ± 33 (257-379)	185 ± 27 (153-230)	–
Vulva – ant. end (VA)	–	–	2470 ± 279 (1959-3038)	1182 ± 129 (910-1397)	–
Vulva – post. end (PV)	–	–	2657 ± 279 (1990-3938)	1062 ± 147 (860-1455)	–
Rectum length	–	–	36 ± 4.6 (30-41)	27 ± 4.1 (19-39)	–
Anal body diam. (ABD)	19.6	17 ± 2.3 (13-22)	47 ± 8.1 (34-58)	35 ± 3.2 (31-41)	11.1 ± 1.3 (8.6-14.1)
Tail with sheath length (T)	–	–	–	–	58 ± 3.1 (52-63)
Tail without sheath length	28	26 ± 3.3 (21-33)	74 ± 8.3 (63-87)	58 ± 8.2 (45-75)	29.4 ± 2.5 (25-34)
Spicule length (SL)	54.1	45 ± 3.7 (38-55)	–	–	–
Gubernaculum length (GL)	18.7	20 ± 2.1 (15-25)	–	–	–
Stoma length/lip region width	1.6	–	1.6 ± 0.3 (1.1-2.1)	1.2 ± 0.2 (0.8-1.7)	–
Nerve ring % (NR/NL × 100)	65.9	69 ± 9.9 (61-96)	65 ± 9.4 (49-86)	67 ± 4.8 (60-82)	–
Excretory pore % (EP/NL × 100)	96.9	98 ± 17 (78-134)	75 ± 11 (51-95)	67 ± 4.8 (60-82)	–
Rectum % (R/ABD × 100)	–	–	79 ± 17 (54-112)	76 ± 13 (52-106)	–
D % (EP /NL × 100)	101.05	109 ± 21 (83-156)	75 ± 11 (55-95)	92 ± 7.9 (80-111)	94 ± 12 (68-120)
E % (EP/T × 100)	343.6	365 ± 68 (236-503)	206 ± 46 (145-303)	197 ± 27 (145-246)	154 ± 14 (128-184)
SW % (SL/ABD × 100)	276	270 ± 50 (170-320)	–	–	–
GS % (GL/SL × 100)	34.56	40 ± 10 (40-60)	–	–	–
H % (H/T × 100	–	–	–	–	47 ± 5.6 (35-56)

**Table 3.** Comparative morphometrics of adult males of *Heterorhabditis ruandica* n. sp., *H. zacatecana* n. sp., and of different closely related *Heterorhabditis* species. All measurements are in µm (except ratios and percentages)

Species	L	BD	EP	NR	NL	T	SL	GL	*a*	*b*	*c*	*c´*	SW%	GS%	D%	Country	Reference
*H. amazonensis*	692–826	36–43	96–116	71–88	97–114	29–41	35–45	19–23	18.7*	7.7**	27.5**	1.3**	120–187	44–56	95–109	Brazil	Andaló et al. (2006)
*H. atacamensis*	842–1025	42–55	116–149	69–93	99–119	24–36	40–49	17–22	19.7*	9.6**	29.3**	1.5**	179–249	38–51	108–126	Chile	Edgington et al. (2011)
*H. bacteriophora*	780–960	38–46	114–130	65–81	99–105	22–36	36–44	18–25	20.8*	9.1*	34.3*	1.8*	174	50	117	Australia	Poinar (1976)
	700–940	37–50	113–140	70–85	95–110	20–27	39–47	18–24	–	–	–	–	–	–	–	Argentina	Agüera de Doucet and Doucet (1986)
	689–880	38–46	78–123	55–90	92–124	21–32	34–48	17–26	–	–	–	1.2*	147–256	41–49	68–106	Australia	Sagun et al. (2015)
	782–927	92–120	103–139	58–76	84–105	28–37	51–53	17–26	6.6–8.5	8.5–10	23–32	1.4–2.2	194–282	37–57	108–157	India	Bhat et al. (2019a)
	805–1075	42–57	84–111	84–75	80–119	24–39	39–51	17–27	16–22	7.1–12	22–41	1.0–1.7	170–225	40–62	77–136	India	Rana et al. (2020)
as *H. argentinensis*^^*#*^^	1000–2000	42–70	145–170	64–82	103–120	28–49	42–49	20–26	16.7*	8.3*	14.3*	1.4*	198*	62*	92*	Argentina	Stock (1993)
as *H. heliothidis*^^*£*^^	1000–1200	32–60	125*	125*	113–131	29–36	42–52	22–27	19–35	8–11	28–38	1.3*	185*	51*	95*	USA	Khan et al. (1976)
*H. baujardi*	818–970	45–53	71–93	54–77	105–132	28–38	33–45	18–22	16–22	6.4–8.8	24–33	1.5**	138–208	44–61	79**	Vietnam	Phan et al. (2003)
	710–903	40–50	83–93	53–68	98–110	33–40	43–48	20–28	16–20	6.7–9.3	18–28	–	154–200	47–61	80–90	India	Vanlalhlimpuia et al. (2018)
as *H. somsookae*^^*#*^^	737–870	37–44	68–93	72–83	90–120	20–30	32–45	17–23	20.7**	8.3**	32.3**	1.2**	133–198	42–59	74–99	Thailand	Maneesakorn et al. (2015)
*H. beicherriana*	889–1192	51–73	130–157	81–108	116–143	32–45	40–49	22–27	15–23	7.2–10	22–34	1.3–2.3	153–208	48–59	102–120	China	Li et al. (2012)
*H. downesi*	699–876	33–40	86–91	62–78	97–106	29–34	41–47	17–19	26.6*	8.8**	27.4**	1.4**	170–220	36–47	90	Ireland	Stock et al., 2002
*H. egyptii*^^*+*^^	594–848	31–56	80–97	56–84	96–109	23–34	25–50	16–22	17.1*	6.6**	19.5**	1.5*	120–220	40–65	84–91	Egypt	Abd–Elgawad and Ameen (2005)
*H. floridensis*	785–294	43–50	104–128	73–90	97–111	29–40	36–46	17–30	19.9*	7.9**	24.1**	1.4**	133–209	47–65	112	USA	Nguyen et al. (2006)
*H. georgiana*	721–913	43–55	101–145	72–93	100–122	29–41	41–49	20–28	16.5*	7.7**	26.1**	1.4**	150–200	51–64	100–122	USA	Nguyen et al. (2008)
*H. hambletoni*^^*+*^^	510–800	38–60	80–100	80–90	–	–	–	–	–	–	–	–	–	–	–	Brazil	Pereira (1937)
*H. hoptha*^^*$*^^	554–837	–	–	–	–	30.9*	43–60	26–30	18–22	5.9–8.2	18–37	1.1*	167**	55**	–	USA	Turco (1970)
*H. indica*	573–788	35–46	109–138	72–85	93–109	24–32	35–48	18–23	17.6*	6.7**	23.0**	1.1**	187	49	121	India	Poinar et al. (1992)
	724–864	41–48	96–113	63–80	89–109	29–36	30–40	21–31	17–20	7.6–8.6	22–27	1.4–1.8	155–210	49–68	101–111	India	Kajol et al. (2020)
	609–916	26–50	78–109	62–83	90–116	18–33	37–48	19–26	16–28	6.5–8.2	25–37	1.0–1.5	116–225	49–64	86–106	India	Bhat et al. (2021b)
as *H. brevicaudis*^^*#*^^	840–950	40–48	92–100	80–88	104–112	28–36	44–48	20–24	–	–	–	2.9*	170*	47*	84*	China	Liu (1994)
as *H. gerrardi*^^*#*^^	508–916	34–48	93–141	54–87	78–115	28–46	34–48	16–27	–	–	–	–	138–274	40–69	100–172	Australia	Plichta et al. (2009)
as *H. hawaiiensis*^^*#*^^	864–1130	49–84	71–146	67–112	100–149	26–40	40–51	18–26	–	–	–	–	–	–	–	USA	Gardner et al. (1994)
as *H. pakistanense*^^*#*^^	720–1013	38–43	112–133	80–110	100–105	30–42	35–42	20–22	18–24	7.2–9.8	19–25	1.4**	144–191	48–65	110–126	Pakistan	Shahina et al. (2017)
*H. marelatus*	805–1046	48–56	110–168	61–95	99–123	24–38	41–49	18–22	15.5*	7.8**	30.0**	1.1**	196	36–50	113**	USA	Liu and Berry (1996)
	960–1010	48–80	107–116	89–95	115–130	37–47	48–52	21–24	–	–	–	–	–	–	–	USA	Stock (1997)
as *H. hepialius*^^*#*^^	8000–1000	65–98	102–131	84–114	113–139	37–49	42–52	17–24	–	–	–	–	–	–	–	USA	Stock et al. (1996)
*H. megidis*	800–1100	44–50	139–176	96–112	122–134	35–43	46–54	17–24	18–22	7–9	23–31	1.6*	188	43	122	USA	Poinar et al. (1987)
*H. mexicana*	614–801	38–47	108–145	61–83	89–108	21–36	30–47	18–32	21.7*	6.8**	27.6**	1.1**	130–196	43–70	114–149	Mexico	Nguyen et al. (2004)
*H. noenieputensis*	530–775	34–46	75–102	64–75	88–106	21–32	37–49	17–24	14–18	5.6–7.9	21–33	1.1–1.7	202–301	38–56	81–108	S. Africa	Malan et al. (2014)
*H. poinari*^^*$*^^	970–1100	43–70	–	–	150–150	36–65	43–55	24–32	95–100	51–95	11–97	–	–	–	–	USA	Kakulia and Mikaia (1997)
***H. ruandica*** **Rw14_NC4a**	**652–863**	**40–51**	**61–109**	**56–74**	**84–117**	**21–29**	**34–50**	**16–23**	**15–21**	**5.8–9.7**	**23–36**	**0.6–1.7**	**150–306**	**35–57**	**61–97**	**Rwanda**	**This study**
*H. safricana*	777–1009	40–58	104–147	52–61	105–126	27–49	35–54	19–27	20.1*	7.9**	43.0**	1.5*	130–259	43–62	92–133	S. Africa	Malan et al. (2008)
*H. taysearae*	648–736	38–48	78–120	54–88	85–123	20–29	30–42	12–21	15.1*	6.5**	14.0**	1.3**	156	46	88	Egypt	Shamseldean et al. (1996)
as *H. sonorensis*^^*#*^^	500–750	32–42	60–84	60–80	80–100	25–45	31–45	20–31	–	–	–	–	110–180	40–75	72–91	Mexico	Stock et al. (2009)
*H. zealandica*	848–1044	36–45	130–150	–	110–128	30–41	48–55	19–25	–	–	–	1.7*	246	44	118	N. Zealand	Poinar (1990)
as *H. heliothidis*^^*#*^^	848–1044	36–45	130–150	–	110–128	30–41	48–55	19–25	–	–	–	1.7*	246	44	118	N. Zealand	Wouts (1979)
***H. zacatecana*** **MEX–39**	**811–914**	**41–56**	**77–109**	**60–78**	**71–108**	**21–33**	**38–55**	**15–25**	**15–25**	**7.6–12**	**26–43**	**1.2–2.5**	**170–320**	**40–60**	**78–134**	**Mexico**	**This study**

**Table 4.** Comparative morphometrics of hermaphrodite females of *Heterorhabditis ruandica* n. sp., *H. zacatecana* n. sp., and of different closely related *Heterorhabditis* species.

Species	L	BD	EP	NR	NL	T	*a*	*b*	*c*	*c´*	V	ABD	D%	Country	Reference
*H. amazonensis*	3517-5587	220-316	184-238	128-171	180-225	104-154	–	–	–	2.3*	42-47	59-83	103*	Brazil	Andaló et al. (2006)
*H. atacamensis*	1791-2904	88-122	165-206	101-132	174-200	72–112	–	–	–	2.7*	39-48	30-46	90-114	Chile	Edgington et al. (2011)
*H. bacteriophora*	3630-4390	160-180	189-217	121-130	189-205	81-93	–	–	–	–	41-47	40-53	106	Australia	Poinar (1976)
	4200-5600	175-242	163-225	125-152	180-220	50-87	–	–	–	–	35-45	45-62	–	Argentina	Agüera de Doucet and Doucet (1986)
	2686-4893	131-241	150-379	80–196	162–302	70-120	–	–	–	1.8*	36-52	43-76	76-126	Australia	Sagun et al. (2015)
	3086-5492	221-352	127-260	79-162	101–200	71-123	9.2-28	23-37	25-75	1.2-3.7	37-52	34-75	112-155	India	Bhat et al. (2019a)
	3916-5155	205-206	153-198	94-127	158-207	70-98	17-21	21-28	46-69	1.7-2.2	37-46	39-51	75-103	India	Rana et al. (2020)
as *H. argentinensis*^^*#*^^	5000-7500	250-575	250-340	132-196	235-300	100–140	–	–	–	1.8*	40-50	70-120	102*	Argentina	Stock (1993)
as *H. heliothidis*^^*£*^^	3000-5100	200-344	250*	250*	163-286	76-100	11-18	11-25	30-63	2.2*	45-52	62.5*	80*	USA	Khan et al. (1976)
*H. baujardi*	3135-4170	180-240	156-192	119-147	186-206	66-114	15-19	16-21	36-50	2.0*	43-48	47-63	88*	Vietnam	Phan et al. (2003)
	3250-3970	190-250	98-115	120-135	180-205	80-105	13-19	16-20	31-45	–	41-49	50-65	73-92	India	Vanlalhlimpuia et al. (2018)
as *H. somsookae*^^*#*^^	2275-3952	108-183	156-214	118-144	158-193	56-87	–	–	–	2.3*	41-56	30-53	86-113	Thailand	Maneesakorn et al. (2015)
*H. beicherriana*	3671-5543	198-374	165-297	135-243	192-343	68-130	13-20	13-25	34-62	1.0-2.3	41-49	51-92	76-94	China	Li et al. (2012)
*H. downesi*	3030-5051	183-291	200-254	175-230	230-244	60-70	–	–	–	1.1*	50-55	57-65	117*	Ireland	Stock et al. (2002)
*H. egyptii*^^*+*^^	2100-3100	107-164	154-205	101-147	144-192	83-115	–	–	–	2.7*	46-59	33-51	104*	Egypt	Abd–Elgawad and Ameen (2005)
*H. floridensis*	3731-5865	217-331	211-301	169-271	271-391	84-126	–	–	–	2.5*	44-49	42-78	104*	USA	Nguyen et al. (2006)
*H. georgiana*	3232-4928	157-267	200-277	143-217	132-271	65-96	–	–	–	1.2*	44-55	42.6*	–	USA	Nguyen et al. (2008)
*H. hambletoni*^^*+*^^	–	–	–	–	–	–	–	–	–	–	–	–	–	Brazil	Pereira (1937)
*H. hoptha*^^*$*^^	–	–	–	–	–	–	–	–	–	–	–	–	–	USA	Turco (1970)
*H. indica*	2300-3100	107-145	163-187	104-123	163-179	72-110	–	–	–	–	45-50	38-51	–	India	Poinar et al. (1992)
	2751-4481	168-273	184-238	115-157	167-204	67-108	16-18	15-25	35-55	1.4-2.5	37-48	30-71	103-132	India	Kajol et al. (2020)
	2861-4227	152-208	140-179	119-146	165-186	79-114	16-23	17-24	30-47	1.5-2.4	39-55	37-56	81-100	India	Bhat et al. (2021b)
as *H. brevicaudis*^^*#*^^	3550-5040	200-312	160-200	144-176	192-240	72-128	–	–	–	–	37-50	56-88	91*	China	Liu (1994)
as *H. gerrardi*^^*#*^^	2049-4288	93-209	103–288	82-210	146-317	90-196	–	–	–	2.4*	40-48	40-80	90-147	Australia	Plichta et al. (2009)
as *H. hawaiiensis*^^*#*^^	4000-7000	270-376	219-318	102-212	187-283	67–98	–	–	–	–	–	38-79	–	USA	Gardner et al. (1994)
as *H. pakistanense*^^*#*^^	1939-4625	102-240	145-186	130-180	155-220	64–95	16–23	11–24	23–58	1.7*	41-49	37-55	68-106	Pakistan	Shahina et al. (2017)
*H. marelatus*	3000-4500	161–233	212-287	133-182	190-244	75-101	–	–	–	1.3*	45-50	20-28	109*	USA	Liu and Berry (1996)
	–	–	–	–	–	–	–	–	–	–	–	–	–	USA	Stock (1997)
as *H. hepialius*^^*#*^^	4000-5200	205-335	175-258	117-161	190-223	60-126	–	–	–	1.9*	45-50	34-60	–	USA	Stock et al. (1996)
*H. megidis*	2400-4900	120-133	193-270	139-178	106-269	95-124	14-24	12-21	23-49	–	45-50	36-86	–	USA	Poinar et al. (1987)
*H. mexicana*	2440-4606	135-267	103-201	114-171	168-221	94-170	–	–	–	2.6*	30-58	40-46	90*	Mexico	Nguyen et al. (2004)
*H. noenieputensis*	2987-5498	168-289	152-209	112-152	166-220	79-120	14-23	18-28	37-58	1.7-3.4	39-47	26-56	77-112	S. Africa	Malan et al. (2014)
*H. poinari*^^*$*^^	1350-2800	54-105	–	–	–	108-112	–	–	–	–	–	–	–	USA	Kakulia and Mikaia (1997)
***H. ruandica*** **Rw14_NC4a**	**2907-4123**	**209-274**	**106-153**	**78-108**	**134-159**	**63-98**	**12-16**	**21-27**	**34-51**	**1.7-2.6**	**45-55**	**29-51**	**67–103**	**Rwanda**	**This study**
*H. safricana*	3373-4073	127-188	210-267	121-163	199-236	64-91	–	–	–	–	43-46	40-54	98-119	S. Africa	Malan et al. (2008)
*H. taysearae*	2200-2800	116-170	137-182	83–120	161-200	72-100	–	–	–	–	40-64	41-67	–	Egypt	Shamseldean et al. (1996)
as *H. sonorensis*^^*#*^^	2856-5799	150-200	115-203	105-180	133-215	122-178	–	–	–	3.0*	50-58	40-75	-	Mexico	Stock et al. (2009)
*H. zealandica*	–	–	–	–	–	–	–	–	–	–	–	–	–	N. Zealand	Poinar (1990)
as *H. heliothidis*^^*#*^^	4000*	247*	–	181*	236	90*	16*	17*	44*	1.7*	46*	53*	–	N. Zealand	Wouts (1979)
***H. zacatecana*** **MEX-39**	**4408-6179**	**235-385**	**108-190**	**96-169**	**174-231**	**63-87**	**13-20**	**20-34**	**52-90**	**1.2-2.4**	**36-57**	**34-58**	**55-95**	**Mexico**	**This study**

**Table 5.** Comparative morphometrics of adult females of *Heterorhabditis ruandica* n. sp., *H. zacatecana* n. sp., and of different closely related *Heterorhabditis* species.

Species	L	BD	EP	NR	NL	T	*a*	*b*	*c*	*c´*	V	ABD	D%	Country	Reference
*H. amazonensis*	1279-2070	70-122	103-126	68-100	119-142	25-38	–	–	–	2.4*	46-50	25-38	–	Brazil	Andaló et al. (2006)
*H. atacamensis*	1754-2628	86-129	154-182	79-119	129-167	80-108	–	–	–	3.8*	43-49	24-33	100-113	Chile	Edgington et al. (2011)
*H. bacteriophora*	3180-3850	160-220	174-214	93-118	155-183	71-93	21.4*	18.8	41.5*	3.1*	42-53	22-31	114	Australia	Poinar (1976)
	1800-2400	100-162	122-162	83-102	108–145	40-65	–	–	–	–	41-50	23-40	–	Argentina	Agüera de Doucet and Doucet (1986)
	1690-3214	100-224	101-212	67-103	120-163	54-101	–	–	–	2.4*	44-50	21-24	72-137	Australia	Sagun et al. (2015)
	1513-2290	84-150	128–181	71-99	113–135	41-79	11-22	11-19	26-42	1.6–2.5	38-51	24-39	108–150	India	Bhat et al. (2019a)
	1226-1819	58-115	108-157	68-91	101-127	29-94	16-25	7.8-16	16-48	1.0-3.4	44-58	24-31	83-116	India	Rana et al. (2020)
as *H. argentinensis*^^*#*^^	2000-3500	90-180	105-240	88-140	162-200	75-108	12.5*	7.8*	31.2*	2.0*	42-48	33-35	100*	Argentina	Stock (1993)
as *H. heliothidis*^^*£*^^	2000-3300	184-240	146*	126*	148-177	71-93	11-15	14-21	26-46	2.8*	48-53	33*	95*	USA	Khan et al. (1976)
*H. baujardi*	1335-2130	90-150	104-149	75-122	131-185	68-89	12-16	10-12	19-32	–	46-51	27-41	–	Vietnam	Phan et al. (2003)
	2060-2290	120-150	98-115	80-95	123-148	78-108	15-17	16-18	20-27	-	41–48	30-38	63-78	India	Vanlalhlimpuia et al. (2018)
as *H. somsookae*^^*#*^^	2159-2666	117-194	143-156	90-112	128-144	41-80	–	–	–	2.9*	36-51	21-35	104-111	Thailand	Maneesakorn et al. (2015)
*H. beicherriana*	1581-3026	125-218	95-165	59-138	105-186	68-105	10-18	10–23	19–34	1.6-2.4	41-49	35–81	88-98	China	Li et al. (2012)
*H. downesi*	1231-2728	74–131	99-126	117-151	111–155	70-122	–	–	–	2.5*	47-60	25-38	–	Ireland	Stock et al. (2002)
*H. egyptii*^^*+*^^	1050-1420	56-84	69-106	69-94	106-125	56-78	17.5**	14.4**	22.2**	3.1**	44-51	19-27	78**	Egypt	Abd–Elgawad and Ameen (2005)
*H. floridensis*	2054-2548	120-156	110-168	86-122	126-178	69-87	–	–	–	–	44-50	32-42	–	USA	Nguyen et al. (2006)
*H. georgiana*	1640-2779	101-188	111-177	96–162	136-219	62-88	–	–	–	1.5*	46-53	42*	–	USA	Nguyen et al. (2008)
*H. hambletoni*^^*+*^^	600-1200	70-100	80-90	70-80	–	–	–	–	–	–	50-58**	–	–	Brazil	Pereira (1937)
*H. hoptha*^^*$*^^	2826-3983	–	148*	161*	219*	28*	13-19	12-21	47-67	0.8*	43–49	33*	92*	New Jersey	Turco (1970)
*H. indica*	1200-1800	76-113	118-138	88–96	120-139	66-88	–	–	–	–	40-53	22-32	–	India	Poinar et al. (1992)
	1713-2242	110-156	135-172	77-92	120-138	61-83	13-17	11-18	22-36	1.9-2.9	44-50	27-33	102-128	India	Kajol et al. (2020)
	1274-1993	70-135	105-129	84-111	124-155	64-83	12-18	10-13	16-31	2.6-4.9	45-52	22-30	77-99	India	Bhat et al. (2021b)
as *H. brevicaudis*^^*#*^^	2100-2500	128-168	124-160	100-108	144-160	76-92	–	–	–	–	45-53	36-48	–	China	Liu (1994)
as *H. gerrardi*^^*#*^^	1428-2533	71-161	108-157	73-141	120-182	66-95	–	–	–	3.3*	43-55	22-38	74-112	Australia	Plichta et al. (2009)
as *H. hawaiiensis*^^*#*^^	1300-2300	104-171	116-175	78-116	110-153	49-87	–	–	–	–	49-56	20-35	–	USA	Gardner et al. (1994)
as *H. pakistanense*^^*#*^^	1413-1785	71-86	130-150	80-100	130-145	65-95	19-21	11-12	16-22	3.1*	44-53	24-27	95*	Pakistan	Shahina et al. (2017)
*H. marelatus*	1600-2600	113-177	139-178	79-119	129–164	55-81	–	–	–	1.3*	45-50	29-48	110*	USA	Liu and Berry (1996)
	–	–	–	–	–	–	–	–	–	–	–	–	–	USA	Stock (1997)
as *H. hepialius*^^*#*^^	3500-4500	99-161	133-177	99-135	150-183	76–113	18*	13*	27*	1.3*	49-51	24-60	88*	USA	Stock et al. (1996)
*H. megidis*	1500-2500	95-140	158-206	105-120	155-168	70-101	15-19	10-16	18-32	2.6*	47-51	25-38	119*	USA	Poinar et al. (1987)
*H. mexicana*	1144-2108	65-123	114-148	76-103	121-150	76-106	–	–	–	–	44-51	21-36	–	Mexico	Nguyen et al. (2004)
*H. noenieputensis*	1075-1697	76-129	102-125	73-90	115-132	63-75	13-17	9-14	17-24	2.3-3.1	40-53	22-32	83-104	S. Africa	Malan et al. (2014)
*H. poinari*^^*$*^^	910-1520	62-80	–	–	152-172	86-105	11-14	50-51	10-11	–	38-50	–	–	USA	Kakulia and Mikaia (1997)
***H. ruandica*** **Rw14_NC4a**	**1131-1608**	**68-83**	**92-129**	**69-97**	**107-132**	**62-88**	**15-20**	**9.0-14**	**16-24**	**1.9–3.6**	**41-51**	**18-34**	**74-104**	**Rwanda**	**This study**
*H. safricana*	1679-2937	102-229	151-196	87–139	148-180	55-111	–	–	–	1.3*	45-50	25-72	97–120	S. Africa	Malan et al. (2008)
*H. taysearae*	830-1400	42-96	120-166	76-109	129-179	62-80	–	–	–	4.0*	44-73	19-28	82*	Egypt	Shamseldean et al. (1996)
as *H. sonorensis*^^*#*^^	1500-2500	85-210	95-140	85-105	129-215	75-99	–	–	–	3.5*	49-53	36-46	93*	Mexico	Stock et al. (2009)
*H. zealandica*	–	–	–	–	–	–	–	–	–	–	–	–	–	N. Zealand	Poinar (1990)
as *H. heliothidis*^^*#*^^	–	–	–	–	–	–	–	–	–	–	–	–	–	N. Zealand	Wouts (1979)
***H. zacatecana*** **MEX-39**	**1954-2798**	**160-228**	**100-133**	**71-96**	**112-148**	**45-75**	**11–15**	**16–21**	**31-63**	**1.3–2.0**	**43-61**	**31–41**	**80-111**	**Mexico**	**This study**

**Table 6.** Comparative morphometrics of infective juveniles of *Heterorhabditis ruandica* n. sp., *H. zacatecana* n. sp., and of different closely related *Heterorhabditis* species.

Species	L	BD	EP	NR	NL	T	*a*	*b*	*C*	*c´*	D%	E%	Country	Reference
*H. amazonensis*	567-612	20-24	89-115	76-93	107-132	98-115	24-29	4.4-5.5	5.1-6.1	7.3*	83-92	89–109	Brazil	Andaló et al. (2006)
*H. atacamensis*	578-666	19-26	101-126	79-101	124-144	94-107	25-31	4.8-5.7	5.7-7.1	5.7*	79-94	149-182	Chile	Edgington et al. (2011)
*H. bacteriophora*	512-671	18-31	87-110	72-93	100-139	83–112	17-30	4.0-5.1	5.7-7.0	6.0*	76-92	103-130	Australia	Poinar (1976)
	530-660	22-30	93-108	80-90	110-130	84-105	23*	4.4*	5.7*	4.8*	81*	106*	Argentina	Agüera de Doucet and Doucet (1986)
	537-587	19-22	87-104	67-83	112-121	94-111	25-31	3.3-3.6	3.6-4.6	7.0*	73-88	87-105	Australia	Sagun et al. (2015)
	474-568	22-28	110-127	61-90	90-115	57-90	19-25	4.7-6.1	5.5-9.3	3.4-7.5	105-139	131-211	India	Bhat et al. (2019a)
	453-167	19-27	72-102	50-74	83-106	47-89	19-29	4.9-7.4	6.0-12	3.7-6.5	78–107	105-189	India	Rana et al. (2020)
*as H. argentinensis*^^*#*^^	610-710	24-38	68-112	82-116	101-150	70-105	18.3*	3.7*	6.5*	4.3*	80*	141*	Argentina	Stock (1993)
*as H. heliothidis*^^*£*^^	619-671	23-29	112*	108*	130-139	104-112	22-28	4.6-5.4	5.8-6.3	6.0*	83*	97*	USA	Khan et al. (1976)
*H. baujardi*	497-595	18-22	91–103	75-86	107-120	83-97	26-30	4.5-5.1	6-6.7	7.2*	78-88	98–114	Vietnam	Phan et al. (2003)
	525-615	18-25	88-96	68-85	98-120	95-108	24-32	4.6-5.9	5.2-6.1	–	74-86	89-92	India	Vanlalhlimpuia et al. (2018)
*as H. somsookae*^^*#*^^	502-565	19-23	81-95	78-94	106-117	91-131	23-27	5-5	4-6	8.0*	76-87	64–95	Thailand	Maneesakorn et al. (2015)
*H. beicherriana*	566-687	21-25	100-122	85-106	118-146	86-111	24–29	4.2-4.9	5.9-6.8	6.0-7.4	80-93	103–121	China	Li et al. (2012)
*H. downesi*	588-692	15-22	96-128	96-105	126-141	62-74	29-42	4.4-5.3	8.5-10.5	4.4*	76-96	160-180	Ireland	Stock et al., 2002
*H. egyptii*^^*+*^^	484-515	18-23	81-94	78-100	100-119	53-75	20-27	4.2-5.2	6.8-9.1	6.9*	74-82	100-170	Egypt	Abd-Elgawad and Ameen (2005)
*H. floridensis*	554-609	19-23	101-122	68-107	123-142	91-113	25-32	3.9-4.9	5.3-6.6	7.2*	71–90	95–134	USA	Nguyen et al. (2006)
*H. georgiana*	547-651	17-26	97-113	74-94	110-139	86-108	23–34	4.1-5.3	5.5-6.9	6.8*	70-93	106	USA	Nguyen et al. (2008)
*H. hambletoni*^^*+*^^	–	–	–	–	–	–	–	–	–	–	–	–	Brazil	Pereira (1937)
*H. hoptha*^^*$*^^	–	–	–	–	–	–	–	–	–	–	–	–	USA	Turco (1970)
*H. indica*	479-573	19-22	88-107	72-85	109-123	93-109	25-27	4.3-4.8	4.5-5.6	–	79-90	83-103	India	Poinar et al. (1992)
	511-546	21-24	92-108	63–73	86-103	24–34	22-25	5-6	4.6-5.4	2.8-5.2	77-96	100-118	India	Kajol et al. (2020)
	516-598	21-25	98-123	82-101	102-129	80-112	24-27	4.5-5.4	4.9-5.7	5.6-8.1	83-97	93–136	India	Bhat et al. (2021b)
as *H. brevicaudis*^^*#*^^	528-632	20-24	104-116	96-104	120-136	68-80	–	–	6.6-8.6	6.3*	81*	150-180	China	Liu (1994)
as *H. gerrardi*^^*#*^^	551-682	18-29	92-111	81-105	110-130	76-141	23-32	16-23	11-21	6.8*	73-92	73-138	Australia	Plichta et al.(2009)
as *H. hawaiiensis*^^*#*^^	506-631	21-28	116-175	79-103	115-181	82-108	–	–	–	6.0*	77*	88*	USA	Gardner et al. (1994)
as *H. pakistanense*^^*#*^^	558-624	19-23	95-106	73-90	113-125	95-110	25-29	4.7-5.3	5.4-6.2	5.4*	78-97	95-107	Pakistan	Shahina et al. (2017)
*H. marelatus*	588-700	24-32	81-113	83-113	121-139	99-117	21-29	4.7-5.4	5.5-6.6	3.0*	60-86	89-110	USA	Liu and Berry (1996)
	567-780	16-24	88-94	71-88	110-119	50-71	31-35	5.1-6.5	10.8-11.5	–	73-88	130-142	USA	Stock (1997)
as *H. hepialius*^^*#*^^	540-600	34-39	84-112	80-101	106-130	49-60	5-7	4-5	9–12	–	79-98	100-200	USA	Stock et al. (1996)
*H. megidis*	736-800	27-32	123-142	104-115	147-160	112–128	23-38	4.6-5.9	6.1-6.9	6.3*	81–91	103-120	USA	(Poinar et al., 1987
*H. mexicana*	530-620	20-24	83-109	74-88	104-142	91-106	24-28	4.2-5.1	5.5-6.3	8.3*	72-86	87-111	Mexico	Nguyen et al. (2004)
*H. noenieputensis*	484-578	21-25	88-105	69-96	79-115	78-95	21-27	4.3-5.2	5.5-6.8	3.4-4.3	81-95	99-125	S. Africa	Malan et al. (2014)
*H. poinari*^^*$*^^	350–410	18-22	–	–	–	15-22	–	–	–	–	–	–	USA	Kakulia and Mikaia (1997)
***H. ruandica*** **Rw14_NC4a**	**496-591**	**18-27**	**70-89**	**52-64**	**103-131**	**49-64**	**20-29**	**4.1-5.4**	**7.6-8.6**	**3.4-5.8**	**66-98**	**112-168**	**Rwanda**	**This study**
*H. safricana*	550-676	19-23	103-122	86-101	125-141	86-108	25-32	3.9-4.9	5.4-7.5	8.7*	80-90	99–133	S. Africa	Malan et al. (2008)
*H. taysearae*	332-499	17-23	74-113	58-87	96–130	44-70	18-27	3.4-4.2	6.5-8.7	3.7*	71-96	110-230	Egypt	Shamseldean et al. (1996)
as *H. sonorensis*^^*#*^^	495-570	19-32	97-116	87-98	110-131	91-125	19-26	4.4-5.4	4.0-6.5	6.7*	78-110	81-111	Mexico	Stock et al. (2009)
*H. zealandica*	570-740	22-30	94-123	90-107	135-147	87-119	25	4.9	6.7	–	73-92	103-109	N. Zealand	Poinar (1990)
as *H. heliothidis*^^*#*^^	570-740	22-30	94-123	90-107	135-147	87-119	25	4.9	6.7	–	73-92	103-109	N. Zealand	Wouts (1979)
***H. zacatecana*** **MEX-39**	**493-578**	**23-27**	**72-99**	**69-72**	**96-124**	**52-63**	**19-24**	**4.4-5.9**	**8.2-10.5**	**4.3–6.7**	**68-120**	**128-184**	**Mexico**	**This study**

### Males

Body 0.65 to 0.86 mm long, C-shaped after fixation. Cuticle almost smooth, with transversal striae poorly developed. Lateral field not visible. Lip region with six lips developed but not fused bearing six acute labial papillae at oral margin and four rounded cephalic papillae at the base of lips. Oral opening almost rounded with thick margins. Amphidial apertures pore-like, ovoid and located posterior to lateral labial papillae. Stoma rhabditoid type, 1.2 to 2.3 times the lip region width, with short cheilostom with poorly refringent rounded cheilorhabdia, short gymnostom with refringent bar-like rhabdia, and long stegostom surrounded by the pharyngeal collar and bearing bar-like pro-mesorhabdia and small poorly refringent meta-telorhabdia. Pharynx poorly developed with robust corpus without differentiated metacorpus, short and slightly narrow isthmus and pyriform bulb with poorly visible valvular apparatus. Nerve ring encircling the isthmus at 58 to 75% of neck length, just anterior to basal bulb. Excretory pore located at basal bulb level, located at 61 to 97% of neck length. Cardia poorly developed, surrounded by intestinal tissue. Intestine without differentiations. Cardiac anterior end with thin walls. Genital system monorchic, laterally reflexed. Spicules well-developed, separate, with small angular manubrium, calamus poorly developed, and robust lamina with acute tip, scarcely prominent dorsal hump and poorly developed ventral velum. Gubernaculum with manubrium straight and slightly ventrally curved corpus, 40 to 50% of spicule length. Tail conoid with acute tip, ventrally curved posteriorly, flanked by the bursa. Bursa peloderan, with nine pairs of genital papillae (1 + 2/3 + 3), one of them probably the phasmid: three pairs pre-cloacal (GP1–GP3) and six pairs post-cloacal being three pairs at mid tail length (GP4–GP6) and three pairs (GP7–GP9) terminal; GP1 and GP2 more spaced, GP2 and GP3 closely spaced (Figs. 1–4).

### Hermaphroditic females

Body 2.91 to 4.12 mm long, arcuate with general morphology similar to male, having labial papillae very acute and prominent. Nerve ring encircling the isthmus at 56 to 78% of neck length. Excretory pore located at or posterior to basal bulb, located at 67 to 103% of neck length. Genital reproductive system didelphic-amphidelphic with ovaries well developed, reflexed, oviducts and uteri not well visible, vagina very short and vulva small having transverse slit opening. Rectum slender, 0.8 to 1.3 times longer than the anal body diameter. Anus with prominent lips. Tail conoid with acute tip lacking mucro, having cellular part simple at its junction with the hyaline part. Phasmids inconspicuous (Figs. 1–4).

### Amphimictic females

Body similar to, but usually smaller than hermaphroditic females, 1.13–1.61  mm long. Rectum very long, almost twice longer than the anal body diameter. Anus with posterior lip very prominent. Tail conoid with acute tip lacking mucro, having cellular part bifurcated at its junction with the hyaline part (Figs. 1–4).

### Infective sheathed juveniles (J3 stage envolved by the J2 stage cuticle)

Body 0.5 to 0.6 mm long, with habitus slightly ventral curved after fixation. Cuticle with transversal striae at anterior end, with both transversal and longitudinal striae at neck region and only with longitudinal striae at rest of body. Lip region lacking differentiate lips, bearing six labial papillae and cephalic papillae not visible. Amphidial apertures very reduced. Oral opening closed, having triradial symmetry. Stoma tubular, about twice the lip region wide. Pharynx slender, with long and narrow corpus, very narrow isthmus and pyriform basal bulb. Nerve ring surrounding the isthmus. Excretory pore at or just posterior to basal bulb. Cardia reduced, surrounded by intestinal tissue. Reproductive system absent. Rectum poorly visible. Anus closed. Tail conoid elongate with acute tip without mucro. Terminal hyaline part 37 to 54% of tail length (Figs. 1–4).

### Infective non-sheathed juveniles (J3 stage)

Body 0.47 to 0.56 mm long, with habitus almost straight after fixation. Cuticle with only transversal striae. Lip region lacking differentiate lips, and labial and cephalic papillae not visible. Oral opening rounded, closed, bearing a large, very refringent dorsal tooth. Amphidial apertures very prominent. Stoma tubular, slightly longer than the lip region wide. Pharynx, nerve ring and excretory pore location similar to the sheathed stage. Cardia reduced, surrounded by intestinal tissue. Rectum poorly visible. Anus closed. Tail conoid with very acute tip without mucro. Terminal hyaline part absent (Figs. 1–4).

### Diagnosis of *Heterorhabditis ruandica* n. sp. and morphological relationships with other species

*Heterorhabditis ruandica* n. sp. is characterized by having hermaphrodite females 2.91 to 4.12  mm long, amphimictic females 1.13 to 1.61  mm long, males 0.65 to 0.86  mm long, and IJs 0.50 to 0.59  mm long. Cuticle with poorly visible annuli in adults, with longitudinal crests in IJ2 and with well-developed annuli in IJ3. Lip region with six low lips having thin and acute lipplets in adults. Lips are poorly developed in IJ2 and bearing a large refringent dorsal tooth in IJ3. Stoma reduced in adults and tubular in IJ. Pharynx robust and short in adults and narrow and slender in IJ. Female reproductive system didelphic–amphidelphic. Anal body diameter in hermaphrodites 29 to 51 µm long, in amphimictic females 18 to 34 µm long, and in males 15 to 25 µm long. Tail short and conoid with acute terminus at cellular part in hermaphrodite females (63-98 µm long, *c*  =  34-51, *c*′  =  1.7-2.6) and slightly bifurcated in amphimictic females (62-88 µm long, *c*  =  16-24, *c*′  =  1.9-3.6). Tail conoid-elongate in IJ2 (49-64  µm long, *c*  =  8.0-12, *c*′  =   3.1-6.2) and IJ3 (22-39  µm long, *c* = 7.6-8.6, *c*′  =  3.4-5.8). Male reproductive system monorchic, with spicules 34 to 50  µm long having reduced manubrium 15 to 21  µm long, bursa peloderan bearing nine pairs of genital papillae (1 + 2/3 + 3).

*Heterorhabditis ruandica* n. sp. is morphologically similar to *H. egyptii*, *H. bacteriophora*, *H. georgiana*, and *H. beicherriana*, and can be distinguished from these species mainly by adult and infective juvenile characters (Tables 3–6). *Heterorhabditis ruandica* n. sp. can be distinguished from *H. egyptii* by the distance from the anterior end to the nerve ring in IJs (52-64 vs. 78-100  µm), the presence of a cephalic tooth in IJs (large vs. apparently small or absent). Additionally, hermaphroditic females of these two species differ in size (2.91-4.12 vs. 2.10-3.10), body diameter (209-274 vs. 107-164  µm), and in the distance from the anterior end to the excretory pore (106-153 vs. 154-205). Amphimictic female of *H. ruandica* n. sp. and *H. egyptii* differ in the size of their tails (62-88 vs. 56-78  µm).

*Heterorhabditis ruandica* n. sp. IJs can be distinguished from the IJs of *H. bacteriophora* by the distance between the anterior end and the excretory pore (67-90 vs. 87-110  µm) and the distance between the anterior end and the nerve ring (52-64 vs. 72-93  µm), and by the tail length (49-65 vs. 83-112  µm). The males of *Heterorhabditis ruandica* n. sp. can be distinguished from the males of *H. bacteriophora* by the distance from the anterior end to the excretory pore (61-109 vs. 114-130  µm) and by the lower D% value (61-97 vs. 117). Hermaphroditic and amphimictic females also show various morphometric differences (Tables 3–6).

*Heterorhabditis ruandica* n. sp. can be distinguished from *H. beicherriana* by the size of IJs (496-591 vs. 566-687  µm), the distance between the anterior end and the excretory pore (67-90 vs. 100-122  µm) and between the anterior end and the nerve ring (52-64 vs. 85-106  µm), and by neck (103-131 vs. 118-146  µm) and tail lengths of IJs (49-65 vs. 86-111  µm). The body length of *H. ruandica* n. sp. males is shorter than the body length of *H. beicherriana* males (652-863 vs 889-1192  µm). Males can also be distinguished by body diameter (40-51 vs. 51-73  µm), and by the distance between the anterior end and the excretory pore (61-109 vs. 130-157  µm) and between the anterior end and the nerve ring (56-74 vs. 81-108  µm), by neck (84-117 vs. 116-143  µm), tail (21-29 vs. 32-45  µm) and gubernaculum (15-21 vs. 22-27  µm) lengths, and by the D% value (61-97 vs. 102-120). Several other morphometric differences were also observed in hermaphroditic and amphimictic females (Tables 3–6).

*Heterorhabditis ruandica* n. sp. can be distinguished from *H. georgiana* by the anterior end to the excretory pore (67-90 vs. 97-113  µm) and the anterior end to the nerve ring (52-64 vs. 74-94  µm) distances, and by the tail length (49-65 vs. 86-108  µm) of IJs. The males can be distinguished by the anterior end to the excretory pore (61-109 vs. 101-145  µm) and the anterior end to the nerve ring (56-74 vs. 72-93  µm) distances, and by tail (21-29 vs. 29-41  µm) and gubernaculum (15-21 vs. 20-28  µm) length, and by D% values (61-97 vs. 100-122). Several other morphometric characters of hermaphroditic and amphimictic females differ between these two species (Tables 3–6).

### Type host and locality

The type hosts are unknown as the nematodes of this genus can be hosted by different insect species and were isolated from soil samples by the *Galleria* baiting technique (Bedding and Akhurst, 1975; White, 1927). Nematode strains *H. ruandica* n. sp. Rw18_M-Hr1a and Rw18_M-Hr1b were collected in the district of Karongi, Western province of the Republic of Rwanda (Decimal degrees coordinates: -2.131500, 29.325467) in a moist habitat along a river bench covered with sweet potato plants. *Heterorhabditis ruandica* n. sp. Rw14_N-C4a nematodes were collected in a ploughed cropland on terraces in a hilly area near Kanyirandori village, Tare sector, Nyamagabe district, Southern province of the Republic of Rwanda (Decimal degrees coordinates: -2.500000, 29.483333).

### Type material

Rw14_N-C4a nematodes are the type material for *Heterorhabditis ruandica* n. sp. Holotype male, and 15 paratype hermaphrodites, males and amphimictic females and 15 third stage juveniles were deposited in the National Nematode Collection of India, IARI, New Delhi, India. Additional specimens were deposited at the nematode collection of the Department of Animal Biology, Plant Biology and Ecology of the University of Jaén, Spain, under the following slide numbers: Rwa001-01 to -12 (25 hermaphrodite females and 6 juveniles), Rwa002-01 to -05 (8 amphimictic females and 9 males), and Rwa003-01 to -02 (8 juveniles). Nematode cultures are maintained in the Institute of Biology, University of Neuchatel, Switzerland and in the Rwanda Agriculture and Animal Resource Development Board, Rubona, Rwanda.

### Etymology

The specific name refers to the country, the Republic of Rwanda (Africa), where the type material, *Heterorhabditis ruandica* n. sp. Rw14_N-C4a nematodes, used to phenotypically characterize the species, were collected.

### *Heterorhabditis zacatecana* n. sp.

**Figure 5:**
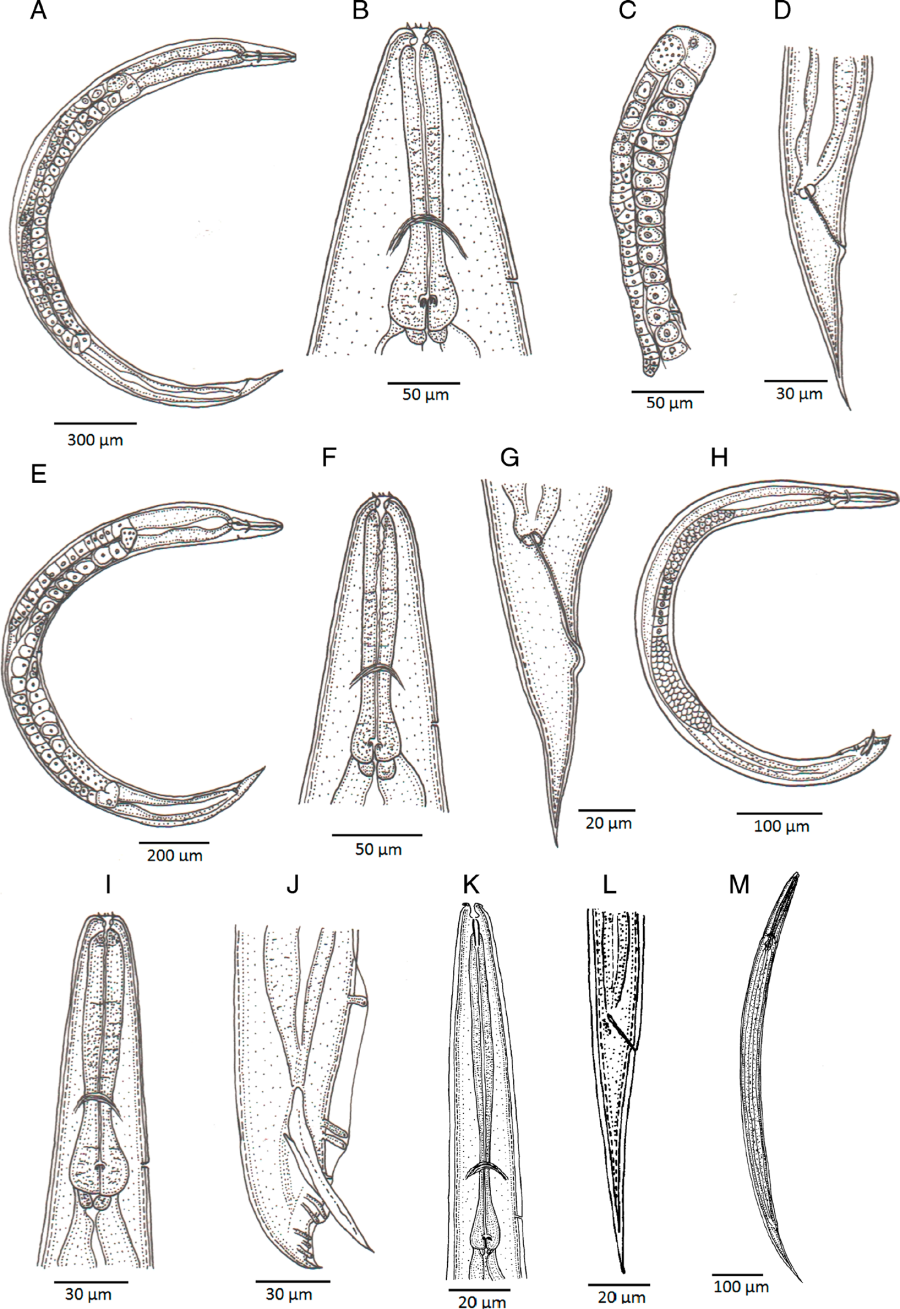
Line drawings of *Heterorhabditis zacatecana* n. sp. (A) A hermaphroditic female. (B) Pharyngeal region of a hermaphroditic female. (C) Anterior part of the reproductive system of a hermaphroditic female. (D) Posterior end of a hermaphroditic female. (E) An amphimictic female. (F) Pharyngeal region of an amphimictic female. (G) Posterior end of an amphimictic female. (H) A male adult. (I) Pharyngeal region of a male adult. (J) Posterior end of a male adult. (K) Pharyngeal region of an infective juvenile. (L) Posterior end of an infective juvenile. (M) An infective juvenile.

**Figure 6:**
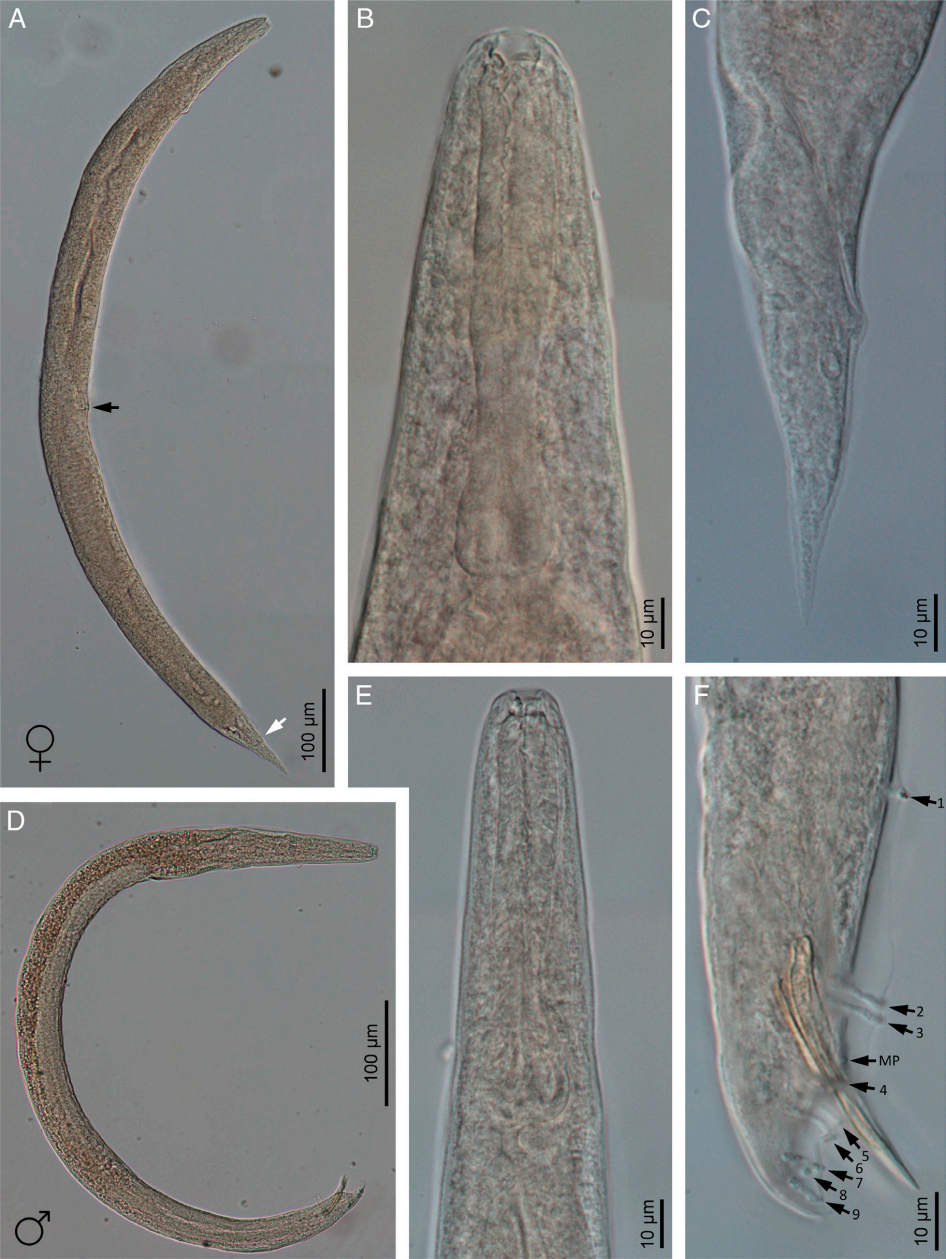
Light microscope micrographs of *Heterorhabditis zacatecana* n. sp. (A) An amphimictic female (black arrow pointing the vulva, white arrow pointing the anus). (B) Pharyngeal region of an amphimictic female. (C) Posterior end of an amphimictic female. (D) A male adult. (E) Pharyngeal region of a male adult. (F) Posterior end of a male adult (arrows pointing at the genital papillae).

**Figure 7:**
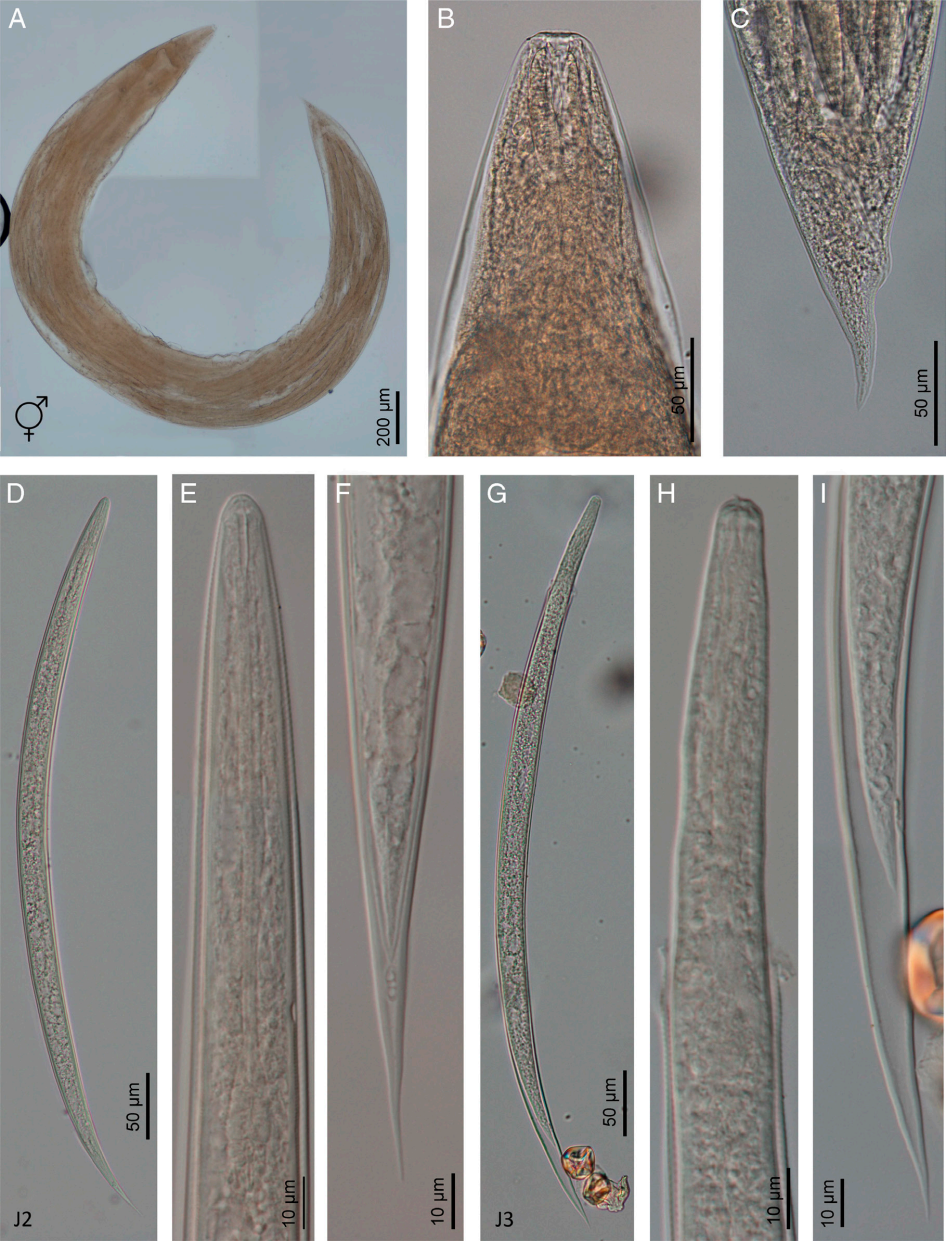
Light microscope micrographs of *Heterorhabditis zacatecana* n. sp. (A) A hermaphroditic female. (B) Pharyngeal region of a hermaphroditic female. (C) Posterior end of a hermaphroditic female. (D) A sheathed third stage juvenile (J2). (E) Pharyngeal region of a sheathed third stage juvenile (J2). (F) Posterior end of a sheathed third stage juvenile (J3). (G) A non-sheathed third stage juvenile (J3). (H) Pharyngeal region of a non-sheathed third stage juvenile (J3). (I) Posterior end of a non-sheathed third stage juvenile.

**Figure 8:**
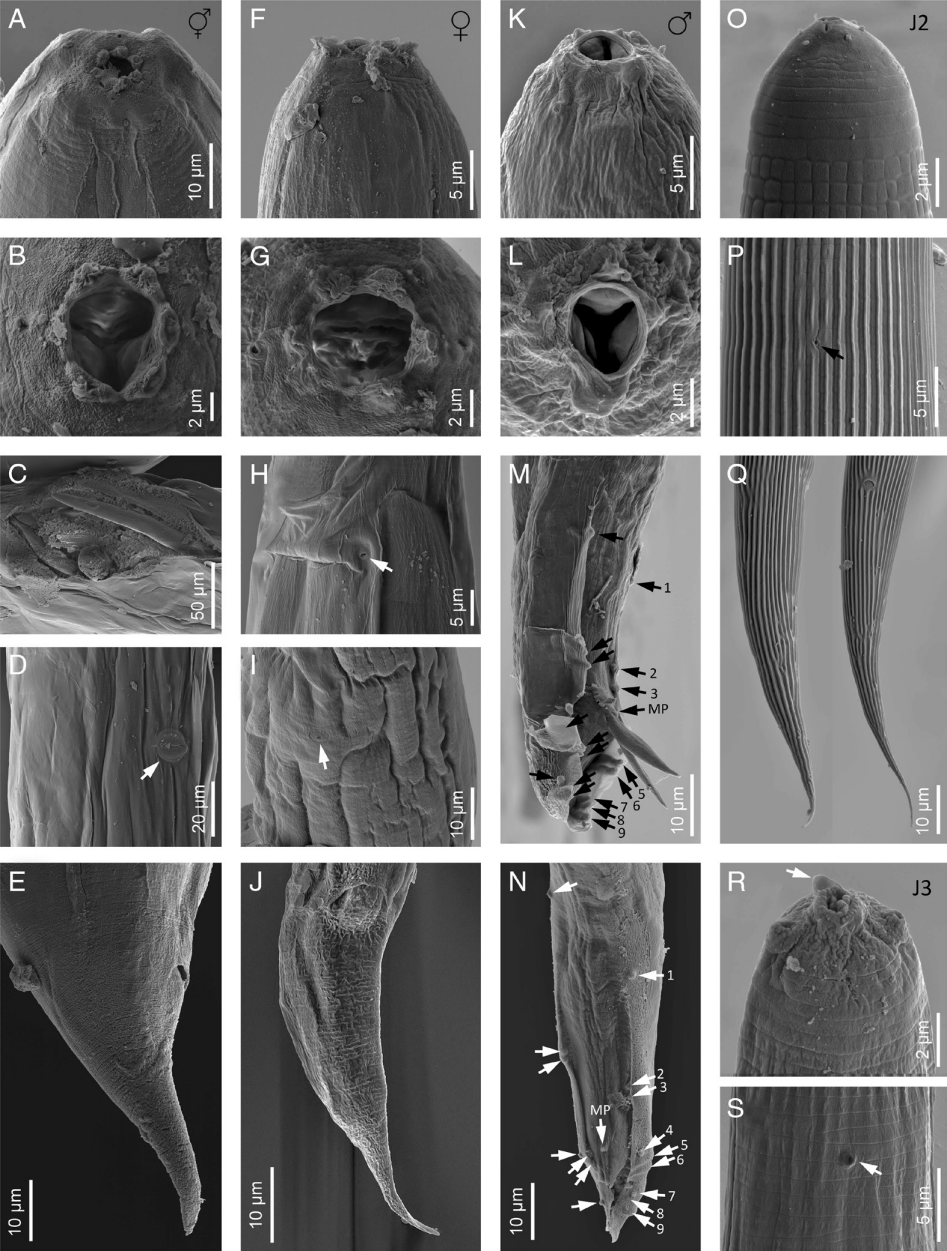
Scanning electron microscope (SEM) micrographs of *Heterorhabditis zacatecana* n. sp. (A, B) Lip region in lateral and frontal views, respectively, of a hermaphroditic female. (C) Broken cuticle of a hermaphroditic female with a juvenile emerging. (D) Vulva of a hermaphroditic female (pointed by a white arrow). (E) Tail of a hermaphroditic female in lateral view. (F, G) Lip region of a female adult in lateral and frontal views, respectively. (H) Excretory pore of a female adult (pointed by a white arrow). (I) Vulva of a female adult. (J) Tail of a female adult in ventral view. (K, L) Lip region of a male adult in lateral and frontal views, respectively. (M, N) Posterior end of a male adult in lateral and ventral views, respectively (arrows pointing at the genital papillae). (O) Lip region of a second-stage juvenile (J2) in lateral view. (P) Cuticle of a second-stage juvenile (J2) (arrow pointing the excretory pore). (Q) Tail of a second-stage juvenile (J2) in lateral and ventral views, respectively. (R) Lip region of a third-stage juvenile (J3) in dorsal view (arrow pointing the frontal tooth). (S) Cuticle of a third-stage juvenile (J3) (arrow pointing the excretory pore).

Figures 5–8, Tables 2 and 3–6

### Males

Body 0.81 to 0.91  mm long, J-shaped after heat killing and body arcuate posteriorly. Cuticle almost smooth, with transversal striae poorly developed. Lateral field not visible. Lip region with six lips poorly developed bearing six acute labial papillae at oral margin and four rounded cephalic papillae at base of lips. Oral opening almost rounded with thick margin. Amphidial apertures pore-like, ovoid and located posterior to lateral labial papillae. Stoma rhabditoid type, 0.9 to 1.6 times the lip region width, with short cheilostom with poorly refringent rounded cheilorhabdia, short gymnostom with refringent bar-like rhabdia, and long stegostom surrounded by the pharyngeal collar and bearing bar-like pro-mesorhabdia and small poorly refringent meta-telorhabdia. Pharynx poorly developed with robust corpus without differentiated metacorpus, short and slightly narrow isthmus and robust pyriform bulb with poorly visible valvular apparatus. Nerve ring encircling the isthmus at 61% to 96% of neck length, just anterior to basal bulb. Excretory pore located at or posterior to the basal bulb, located at 78% to 134% of neck length. Cardia poorly developed, surrounding by intestinal tissue. Intestine without differentiations. Genital system monorchic, laterally reflexed. Spicules well developed, separate, with more or less rounded manubrium, calamus poorly developed, and thinner and slender lamina with acute tip, scarcely prominent dorsal hump and poorly developed ventral velum. Gubernaculum with manubrium slightly ventral curved and straight corpus, 40% to 60% of spicule length. Tail conoid with acute tip, ventrally curved posteriorly, flanked by the bursa. Bursa peloderan, with nine pairs of genital papillae (1 + 2/3 + 3), one of them probably the phasmid: three pairs pre-cloacal (GP1–GP3) and six pairs post-cloacal being three pairs at mid tail length (GP4–GP6) and three pairs (GP7–GP9) terminal; GP1 and GP2 more spaced, GP2 and GP3 closely spaced (Figs. 5–8).

### Hermaphroditic females

Body 4.41 to 6.18  mm long, arcuate with general morphology similar to male, having labial papillae more acute and prominent. Genital reproductive system didelphic–amphidelphic with ovaries well developed, reflexed, oviducts and uteri not well visible, vagina very short and vulva small having transverse slit opening. Rectum slender, about 1.5 times longer than the anal body diameter. Anus with prominent lips. Tail conoid with acute tip lacking mucro, having cellular part simple at its junction with the hyaline part. Phasmids inconspicuous (Figs. 5–8).

### Amphimictic females

Body similar to, but usually smaller than hermaphrodites, 1.95 to 2.80  mm long. Rectum very long, about twice longer than the anal body diameter. Anus with posterior lip more prominent. Tail conoid with acute tip lacking mucro, having cellular part simple at its junction with the hyaline part (Figs. 5–8).

### Infective sheathed juveniles (J3 stage envolved by the J2 stage cuticle)

Body 0.49–0.58  mm long, with habitus slightly ventral curved after fixation. Cuticle with transversal striae at anterior end, with both transversal and longitudinal striae at neck region and only with longitudinal striae at rest of body. Lip region lacking differentiate lips, bearing six labial papillae and cephalic papillae not visible. Amphidial apertures very reduced. Oral opening closed, having triradial symmetry. Stoma tubular, about twice the lip region wide. Pharynx slender, with long and narrow corpus, very narrow isthmus and pyriform basal bulb. Nerve ring surrounding the isthmus. Excretory pore at or just posterior to basal bulb. Cardia reduced, surrounded by intestinal tissue. Reproductive system absent. Rectum poorly visible. Anus closed. Tail conoid elongate with acute tip without mucro. Terminal hyaline part 31% to 56% of tail length (Figs. 5–8).

### Infective non-sheathed juveniles (J3 stage)

Body 0.47 to 0.55  mm long, with habitus slightly ventral curved after fixation. Cuticle with only transversal striae. Lip region lacking differentiate lips, and labial and cephalic papillae not visible. Oral opening rounded, closed, bearing a small dorsal tooth. Amphidial apertures very prominent. Stoma tubular, slightly longer than the lip region wide. Pharynx, nerve ring and excretory pore location similar to the sheathed stage. Cardia reduced, surrounded by intestinal tissue. Rectum poorly visible. Anus closed. Tail conoid with acute tip without mucro. Terminal hyaline part absent (Figs. 5–8).

### Diagnosis of *Heterorhabditis zacatecana* n. sp. and relationships with other species

*Heterorhabditis zacatecana* n. sp. is characterized by having hermaphrodite females 4.41 to 6.18  mm long, amphimictic females 1.9 to 2.7  mm long, males 0.81 to 0.91  mm long, and IJs 0.49 to 0.57  mm long. Cuticle with poorly visible annuli in adults, with longitudinal crests in IJ2 and with well-developed annuli in IJ3. Lip region with six low lips having thick and acute lipplets in adults. Lips are poorly developed in IJ2 and bearing a small refringent dorsal tooth in IJ3. Stoma reduced in adults and tubular in IJs. Pharynx robust and short in adults, and narrow and slender in IJs. Female reproductive system didelphic–amphidelphic. Anal body diameter in hermaphrodites 34 to 58  µm long, in amphimictic females 31 to 41 µm long, and in males 13 to 22  µm long. Tail short and conoid with acute terminus at cellular part in hermaphrodite females (63-87  µm long, *c*  =  52-90, *c*′  =  1.2-2.4). and in amphimictic females (45-75  µm long, *c*  =  31-63, *c*′  =  1.3-2.0). Tail conoid-elongate in IJ2 (52-63  µm long, *c* = 7.9–9.8, *c*′  =  4.0-6.5) and in IJ3 (25-34  µm long, *c* = 8.2-10.5, *c*′  =  4.3-6.7). Male reproductive system monorchic, with spicules 38 to 55  µm long having conoid manubrium 15 to 25  µm long, bursa peloderan bearing nine pairs of genital papillae (1 + 2/3 + 3).

*Heterorhabditis zacatecana* n. sp. is morphologically similar to *H. ruandica* n. sp.*, H. amazonensis*, *H. bacteriophora*, *H. georgiana* and *H. beicherriana*, and can be distinguished from these species mainly by adults and infective juvenile characters (Tables 3–6). *Heterorhabditis zacatecana* n. sp. can be distinguished from *H. ruandica* n. sp., one of the morphologically most similar species, by the shape of the male spicule (slender vs. robust) and the manubrium size (large vs. small), the size of hermaphrodites (4.41-6.18 vs. 2.91-4.12  mm), the hermaphrodite neck length (174-231 vs. 134-159  µm), and the hermaphrodite c ratio (52-90 vs. 34-51). The size of amphimictic females (1.95-2.80 vs. 1.13-1.61  µm), the shape of the tail tip (acute and longer vs. with mucro), the type of cellular–hyaline junction part (simple vs. bifurcated), the body diameter (160-228 vs. 68-83  µm), the a (11-15 vs. 15-20), b (16-21 vs. 9-14), and c ratios (31-63 vs. 16-24) and the anal body diameter (31-41 vs. 18-34  µm) differ also between *H. zacatecana* n. sp. and *H. ruandica* n. sp. IJs anterior ends also differ between these two species (small vs. large), and the presence of a cephalic tooth (small or absent vs. refringent and large).

Morphologically, the IJs of *H. zacatecana* n. sp. can be distinguished from the IJs of *H. amazonensis* by their size (493-578 vs. 567-612  µm), the distance from the anterior end to the nerve ring (59-72 vs. 76-93  µm), the neck length (96-124 vs. 107-132  µm), the tail length (52-63 vs. 98-115 µm), the a (19-24 vs. 24-29), c (8.2-10.5 vs. 5.1-6.1), and c′ (4.3-6.7 vs. ca. 7.3  µm) ratios and the E% (128-184 vs. 89-109). Moreover, hermaphroditic females differ in body size (4.41-6.12 vs. 3.52-5.59  mm), tail length (62-87 vs. 104-154  µm) and anal body diameter (34-58 vs. 59-85  µm). Amphimictic females of these two species differ also in body size (1.95-2.80 vs. 1.28-2.07  µm), tail length (45-75 vs. 25-38  µm), and body diameter (160-228 vs. 70-122). Male sizes differ between *H. zacatecana* n. sp. and *H. amazonensis* (0.81-0.91 vs. 0.69 vs. 0.83  mm) and body diameter (41-56 vs. 36-43  µm).

*Heterorhabditis zacatecana* n. sp. IJ can be distinguished from *H. bacteriophora* by the distance from the anterior end to the nerve ring (59-72 vs. 72-93  µm), and the tail length (52-63 vs. 83-112  µm). In the case of males, they differ in the distance from the excretory pore to the anterior end (77-109 vs. 114-130  µm) and in body diameter (41-56 vs. 38-46  µm). Several morphometric differences were also observed in hermaphrodites and amphimictic females (Tables 2–6).

*Heterorhabditis zacatecana* n. sp. IJs can be distinguished from *H. beicherriana* IJs by the distance from anterior end to the excretory pore (72-99 vs. 100-122  µm) and the distance from the anterior end to the nerve ring (59-72 vs. 85-106  µm), the tail length (52-63 vs. 86-111), values of a (19-24 vs. 24-29), *c*´ (4.3-6.7 vs. 6.0-7.4), and c (8.2-10.5 vs. 5.9-6.8) ratios, and the E% value (128-184 vs. 103-121). Males can be differentiated by differences in neck (71-108 vs. 116-143  µm) and tail (21-33 vs. 32-35  µm) lengths, the distance from the anterior end to the excretory pore (77-109 vs, 130-157  µm) and from the anterior end to the nerve ring (60-78 vs. 81-100  µm). Several morphometric differences were also observed in hermaphrodites and amphimictic females of these two species (Tables 2–6).

*Heterorhabditis zacatecana* n. sp. IJs can be distinguished from *H. georgiana* IJs by differences in body diameter (23-27 vs. 17-26  µm), tail length (52-63 vs. 86-108  µm), and anterior end to excretory pore (72-99 vs. 97-113  µm) and anterior end to nerve ring distances (59-72 vs. 74-94  µm). The a, b and c ratios, E% and D% of IJs differ also in these two species. The males of these two species differ in anterior end to excretory pore (77-109 vs. 101-145  µm) and anterior end to nerve ring distances (60-78 vs. 72-93  µm), and neck (71-108 vs. 100-122  µm) and tail (21-33 vs. 29-41  µm) lengths. Several morphometric characters of hermaphroditic and amphimictic females differ between these two species (Tables 2–6).

### Type host and locality

The type hosts are unknown as the nematodes of this genus can be hosted by different insect species and were isolated from soil samples by the *Galleria* baiting technique (Bedding and Akhurst, 1975; White, 1927). *Heterorhabditis zacatecana* n. sp. MEX-39 and MEX-40 nematodes were collected in maize fields in Villanueva (Zacatecas, Mexico; decimal degrees coordinates: 22.161371, -102.887940), and *Heterorhabditis zacatecana* n. sp. MEX-41 nematodes were collected in maize fields in Apaseo el Alto (Guanajuato, Mexico; decimal degrees coordinates: 20.470774, -100.59571).

### Type material

MEX-39 nematodes are the type material for *Heterorhabditis zacatecana* n. sp. Holotype male, 15 paratype and 15 third stage juveniles were deposited in the National Nematode Collection of India, IARI, New Delhi. Additional specimens were deposited in the nematode collection of the Department of Animal Biology, Plant Biology and Ecology of the University of Jaén, Spain, under the following slide numbers: Mex001-01 to -03 (6 hermaphrodite females), Mex002-01 to -04 (8 amphimictic females and 3 males), and Mex003-01 to -04 (14 juveniles). Nematode cultures are maintained in the Institute of Biology, University of Neuchatel, Switzerland.

### Etymology

The specific name refers to the Mexican state, Zacatecas, where the type material, *Heterorhabditis zacatecana* n. sp. MEX-39 nematodes, used to phenotypically characterize the species were collected.

### Cross-hybridization experiments

No progeny was observed when males and females of *H. ruandica* n. sp. Rw14_N-C4a and of *H. zacatecana* n. sp. MEX-39 were left to interact. No progeny was observed when males and females of *H. ruandica* n. sp. Rw14_N-C4a and of *H. bacteriophora* CH21 were left to interact. No progeny was observed when males and females of *H. zacatecana* n. sp. MEX-39 and of *H. bacteriophora* CH21 were left to interact. When males and females of *H. ruandica* n. sp. Rw14_N-C4a were crossed, fertile progeny was observed. When males and females of *H. zacatecana* n. sp. MEX-39 were crossed, fertile progeny was observed. When males and females of *H. bacteriophora* CH21 were crossed, fertile progeny was observed. Similarly, *H. zacatecana* n. sp. MEX-39 and *H. zacatecana* n. sp. MEX-40 nematodes produced fertile progeny, and *H. ruandica* n. sp. Rw18_M-Hr1a and *H. ruandica* n. sp. Rw14_N-C4a nematodes produced fertile progeny. These results provide further support for the heterospecific status of the Rwandan and the Mexican nematode populations.

### Nematode molecular characterization and phylogenetic relationships

**Figure 9:**
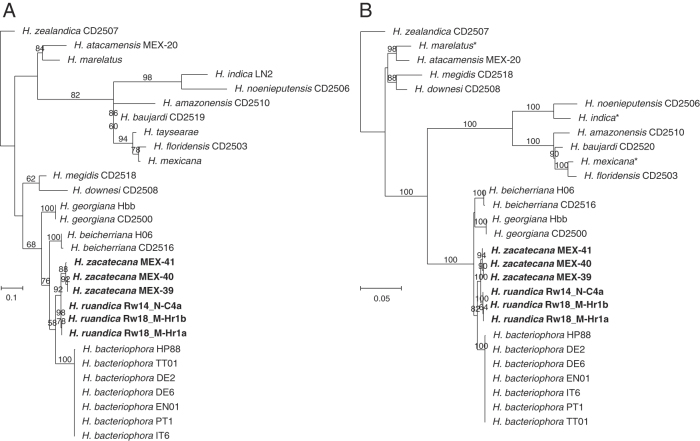
Maximum-likelihood phylogenetic tree reconstructed from: (A) the sequences of the cytochrome c oxidase I (*COI*) of different *Heterorhabditis* species. A total of 343 nucleotide positions, flanked by primers HCF and HCR, were analyzed; and (B) the concatenated sequences of the following genes/genetic regions of different *Heterorhabditis* species: the D2–D3 expansion segments of the 28S rRNA (*D2–D3*), the internal transcribed spacer (*ITS*) of the rRNA (ITS), and the cytochrome c oxidase I (*COI*). A total of 1673 concatenated nucleotide positions were included in the reconstruction. Accession numbers of the nucleotide sequences used for the analyses are shown in Table S3. *For *H. marelatus*, *H. indica*, *and H. mexicana,* the sequences that were concatenated are derived from different nematode isolates. *Heterorhabditis safricana*, and *H. tayserae* were not included as their *COI* or their D2–D3 sequences, respectively, are not publicly available. Numbers at nodes represent bootstrap values based on 100 replications. Bars represent average nucleotide substitutions per sequence position.

**Figure 10:**
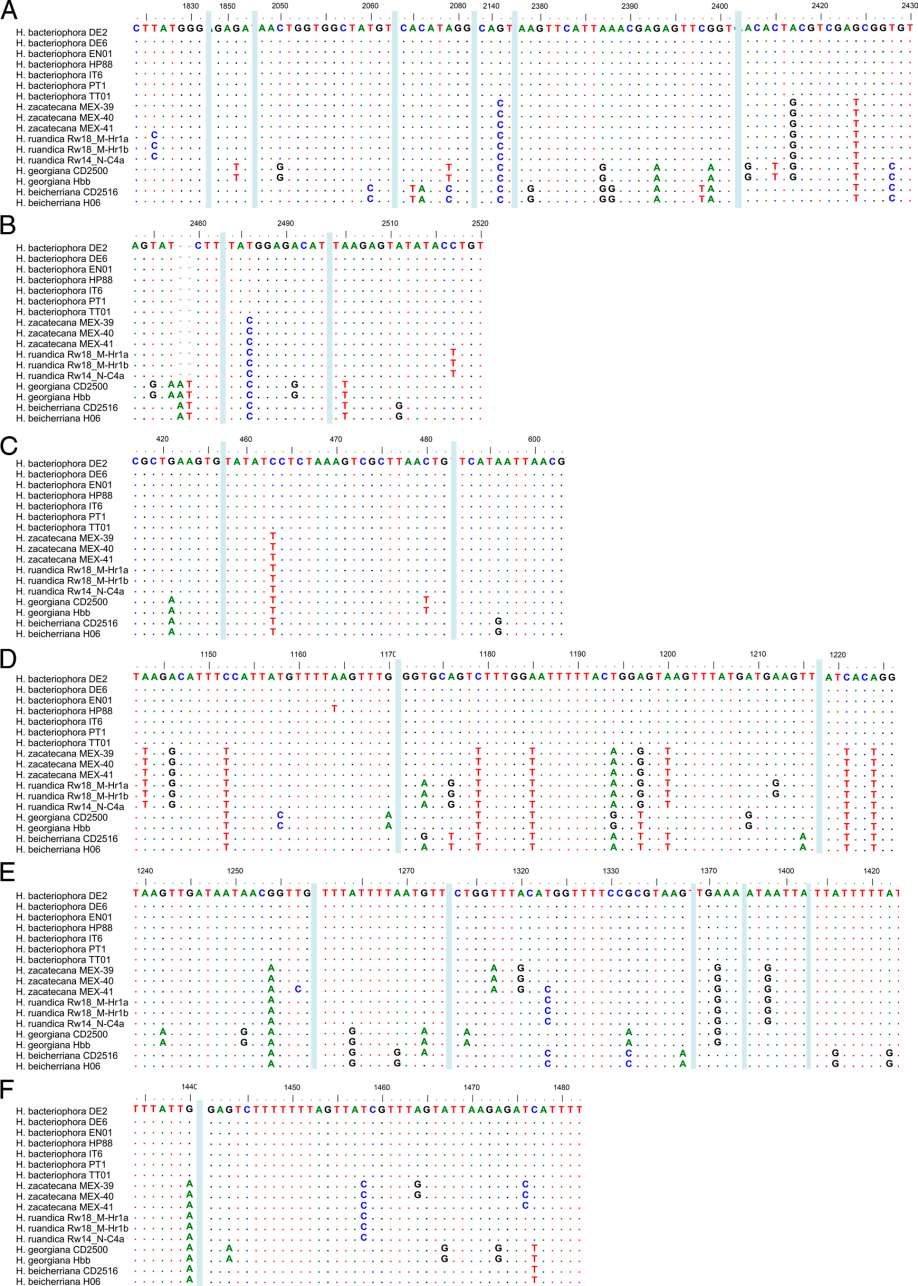
Polymorphism in the sequences of the ITS region (A, B), the D2–D3 region (C), and the *COI* gene (D-F) showing taxonomically relevant nucleotide positions for *Heterorhabditis* nematodes of the “*Bacteriophora-*group”. Nucleotide position numbers of rRNA genes are according to the sequences of *C. elegans* N2 (NCBI accession number: MN519140) and of mitochondrial genes are according to the sequences of *C. elegans* N2 (NCBI accession number: AY171203).

**Figure 11:**
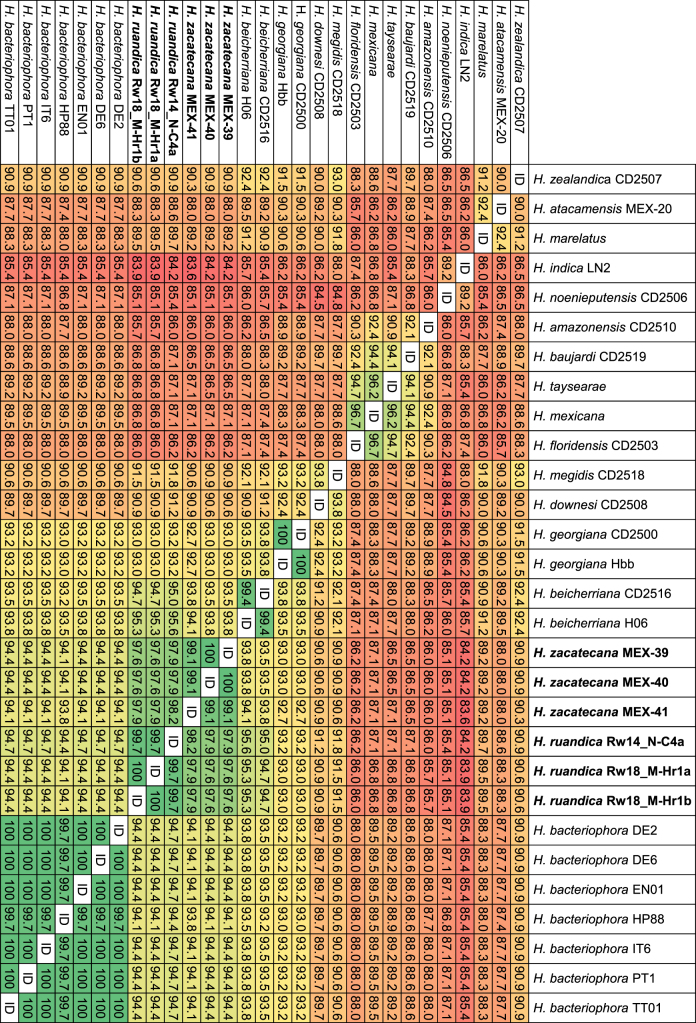
Pairwise nucleotide similarities (%) in the sequences of the cytochrome c oxidase I (*COI*) gene of different *Heterorhabditis* species. A total of 344 nucleotide positions, flanked by primers HCF and HCR, were analyzed. Accession numbers of gene sequences used are shown in Table S3.

Phylogenetic reconstructions based on nuclear and mitochondrial genes (*ITS*, *D2–D3*, *COI*, *umc-87*, and *cmd–1*), either individually or concatenated, confirm that the nematodes of the genus *Heterorhabditis* are grouped into three major clades: the “*Megidis*-group”, the “*Indica*-group” and the “*Bacteriophora*-group”, which is consistent with previous studies (Dhakal et al., 2020) (Fig. 9, Fig. S1). The clade of the “*Bacteriophora*-group” is, in turn, separated into five subclades. Three of them are composed of already described species: *H. beicherriana*, *H. georgiana*, and *H. bacteriophora*, and two of them are composed of two new, undescribed species, which we named here *H. zacatecana* n. sp., and *H. ruandica* n. sp. (Fig. 9, Fig. S1). Clearer phylogenetic separations within the species of the clade of the “*Bacteriophora*–group” were observed when phylogenies were reconstructed based on *COI*, *ITS,* or on concatenated sequences of *COI, ITS,* and D2–D3 (Fig. 9, Fig. S1). Closer inspection at the *ITS*, D2–D3 and *COI* sequences reveals unambiguous genetic differences between the nematodes of the “*Bacteriophora*–group” (Fig. 10). Sequence similarity scores and nucleotide difference counts show a closer relationship between *H. bacteriophora*, *H. ruandica* n. sp., and *H. zacatecana* n. sp. nematodes (Fig. 11 and Figs. S2-S6). *Heterorhabditis ruandica* n. sp. and *H. bacteriophora* share 99.1% and differ in 6 nucleotide positions in the ITS sequences flanked by primers TW81 and AB28, share 99.8% and differ in 1 nucleotide position in the D2–D3 sequences flanked by primers D2A and D3B, and share 94.1 to 94.7% and differ in 18 to 19 nucleotide positions in the *COI* sequences flanked by primers HCF and HCR (Fig. 11 and Figs. S2-S6). *Heterorhabditis zacatecana* n. sp. and *H. bacteriophora* share 99.4% and differ in 4 nucleotide positions in the *ITS* sequences flanked by primers TW81 and AB28, share 99.8% and differ in 1 nucleotide position in the D2–D3 sequences flanked by primers D2A and D3B, and share 94.1 to 94.4% and differ in 19 to 20 nucleotide positions in the *COI* sequences flanked by primers HCF and HCR (Fig. 11 and Figs. S2-S6). *Heterorhabditis ruandica* n. sp. and *H. zacatecana* share 99.7% and differ in 2 nucleotide positions in the *ITS* sequences flanked by primers TW81 and AB28, share 100% and differ in no nucleotide position in the D2–D3 sequences flanked by primers D2A and D3B, and share 97.6% to 98.2% and differ in 6–8 nucleotide positions in the *COI* sequences flanked by primers HCF and HCR (Fig. 11 and Figs. S2-S6). Noteworthy, we observed almost no intraspecific variations within the nematodes of the “*Bacteriophora*-group” at different genetic loci (Figs. 10, 11, and Figs. S2–S6). However, the sequences of the *COI* gene show very interesting signatures of population–specific polymorphism (Figs. 10D-F, 11). Specifically, *Heterorhabditis ruandica* n. sp. Rw18_M-Hr1a and Rw18_M-Hr1b nematodes that were collected in the same western Rwandan region differ from the *Heterorhabditis ruandica* n. sp. Rw14_N-C4a nematodes collected in a southern Rwandan region in a transitional nucleotide change (g.1212A > G) (Fig. 10D). Moreover, *H. zacatecana* n. sp. MEX-39 and MEX-40 nematodes collected in north-central Mexico and *H. zacatecana* n. sp. MEX-41 nematodes collected in central Mexico differ in three transitional nucleotide changes (g.1257T > C, g.1324T > C, and g.1464A > G) (Fig. 10D-F). Hence, due to its highly conserved species–specific polymorphism, and the consistent population-specific polymorphic patterns, the *COI* gene emerges as an important phylogenetic marker also for the genus *Heterorhabditis*, in a similar manner as it is for many other taxonomic groups (Hebert et al., 2003; Pentinsaari et al., 2016).

### Interspecific genetic variability within the *H. bacteriophora* clade

In a recent study, Dhakal et al. (2020) studied several hundreds of ITS sequences of *Heterorhabitis* nematodes and recognized that nematodes identified as *H. bacteriophora* are represented by at least three haplotypes, some of which, the authors hypothesized, could represent new species. We contrasted their results and the sequences they used with the sequences we generated and found out that *H. bacteriophora* DE2, DE6, EN01, HP88, IT6, and PT1 nematodes represent Dhakal’s haplotype 1 (Figs. S7 and S8). Dhakal’s haplotype 2 is actually a mixture of two haplotypes: one represented by *H. zacatecana* n. sp. MEX-39, MEX-40, MEX-41, and by strains N2 and MK with identical ITS sequences; and one represented by strains UP2A2, 267, 269, 270, 271, 275, and 276 with identical ITS sequences. Strains MEX-39, MEX-40, MEX-41, N2 and MK differ in a transitional nucleotide change (g.2049A > G) with strains UP2A2, 267, 269, 270, 271, 275, and 276. Hence, strains N2, MK are likely *H. zacatecana*, and strains UP2A2, 267, 269, 270, 271, 275, and 276 might represent a new species. However, full characterization is needed to prove this hypothesis. Dhakal’s haplotype 3, represented by NGPS20, among others isolates, might also represent a new species, but again full characterization is needed to prove this hypothesis. In addition, our analyses reveal what we call a fourth haplotype, to follow Dhakal’s system, which is represented by *H. ruandica* Rw18_M-Hr1a, Rw18_M-Hr1b, and Rw14_N-C4a nematodes (Figs. S7 and S8). Phylogenetic reconstructions show a clear phylogenetic separation between all these haplotypes (Fig. S8). Hence, some of the haplotypes described by Dhakal et al. (2020) represent new species, closely related to *H. bacteriophora*, and some others likely represent new species, which highlights the power of statistical parsimony network analyses to uncover undescribed species of the genus *Heterorhabditis*, and supporting previous hypothesis regarding the taxonomic status of these nematode isolates (Bruno et al., 2020; Dhakal et al., 2020; Fallet et al., 2020).

### Symbiotic relationships

**Figure 12:**
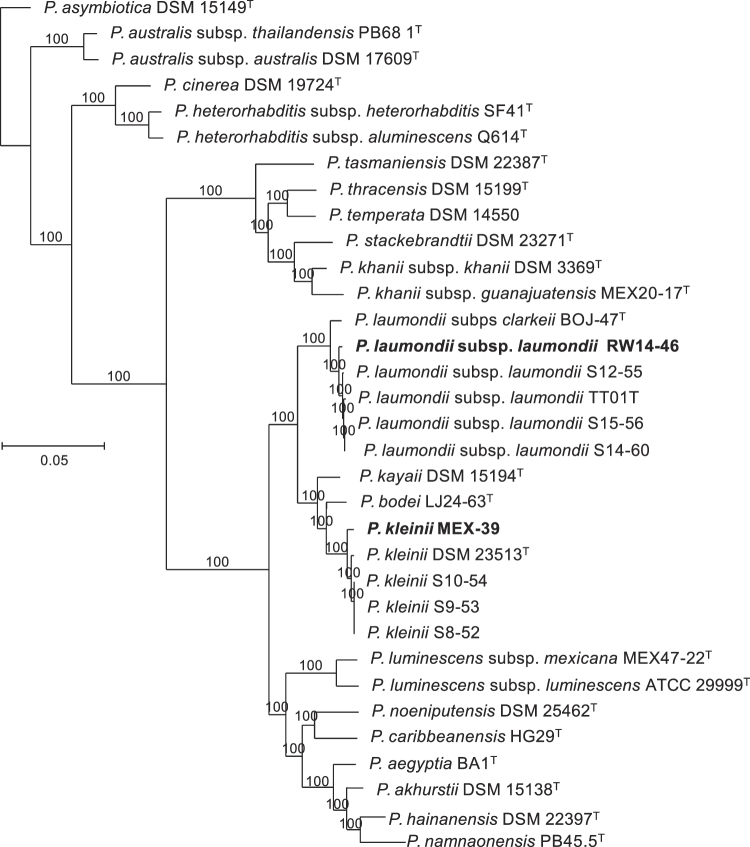
Phylogenetic reconstruction based on core genome sequences of *Photorhabdus* bacterial strains. Numbers at the nodes represent SH-like branch supports. Bar represents average nucleotide substitutions per sequence position. Accession numbers of the genome sequences used for the reconstruction are shown in Table S4.

Up to now, the bacterial genus *Photorhabdus* Boemare, Akhurst and Mourtant 1993 contains 27 taxa, including species and subspecies (Machado et al., 2021b). Phylogenetic relationship reconstructions based on whole genome sequences show that the bacterial symbionts isolated from *H. zacatecana* n. sp. MEX-39 and *H. ruandica* n. sp. Rw14_N-C4a nematodes, named here as MEX-39 and RW14-46, respectively, show high similarity with two of the already described *Photorhabdus* species: *Photorhabdus kleinii* and *P. laumondii* subsp. *laumondii*, respectively (Fig. 12). *Photorhabdus kleinii* MEX-39 shares 87–88% digital DNA–DNA hybridization (dDDH) with other members of the same species, while *P. laumondii* subsp. *laumondii* RW14-46 shares 89% digital DNA–DNA hybridization (dDDH) with other members of the same species, (Fig. S9).

### On the synonymization and declaration of *species inquirendae* of some species

**Figure 13:**
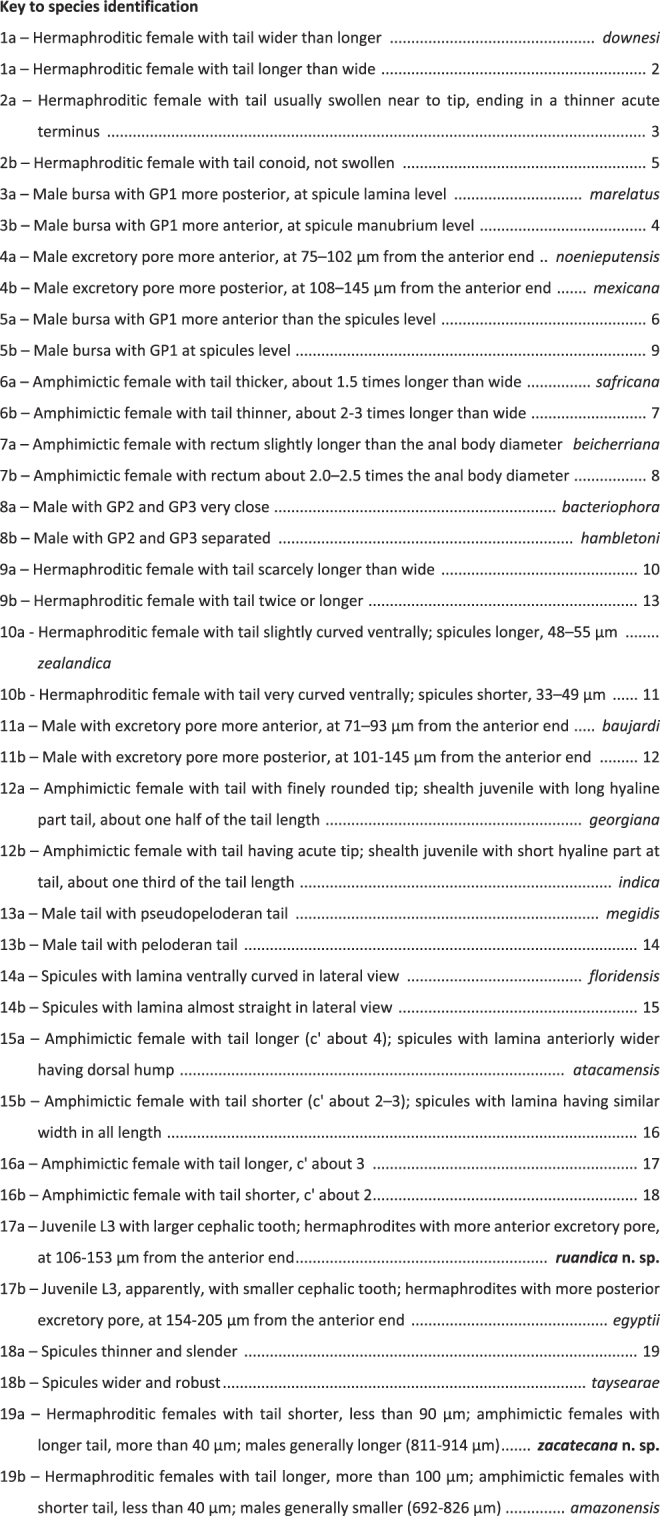
Dichotomous key to identify the species of the genus *Heterorhabditis* based on morphological and morphometrical characters of L3 juveniles, of male and female adults, and of hermaphroditic females.

We revised the original publications of all synonymized species and based on their morphology and molecular data (when available), we reinforce the synonymized status of most of them (Khan et al., 1976; Wouts, 1979; Stock, 1993; Gardner et al., 1994; Liu, 1994; Stock et al., 1996; Plichta et al., 2009; Stock et al., 2009; Maneesakorn et al., 2015; Hunt and Nguyen, 2016; Shahina et al., 2017; Dhakal et al., 2020). However, the original description of *H. bacteriophora* provided by Poinar (1976) shows males with very anterior GP1 while in its synonymized species *H. heliothidis* (Khan, Brooks & Hirschmann, 1976) Poinar, Thomas & Hess, 1977 (=*Chromonema heliothidis* Khan et al., 1976) the GP1 appears more posterior (Khan et al., 1976; Poinar, 1976). Hence, it is likely that both species are not conspecific. Therefore, we declare *H. heliothidis* (Khan, Brooks & Hirschmann, 1976) Poinar, Thomas & Hess, 1977 as *species inquirenda*. *Heterorhabditis hoptha* and *H. poinari* were poorly described (Turco, 1970; Kakulia and Mikaia, 1997). Original descriptions lack differentiated description of all diagnostic characters of adult and larval stages. According to this, both species should remain in the list of *species inquirendae*. *Heterorhabditis egyptii* and *H. hambletoni* were described showing all diagnostic characters of adults and larvae stages. According to this, both species are considered valid herein (Pereira, 1937; Abd-Elgawad and Ameen, 2005). The lack of molecular data, however, impairs their inclusion in future phylogenetic studies. Nevertheless, new species description should contrast morphological characters with these species. An updated dichotomous key to identify the species of the genus *Heterorhabditis* is provided (Fig. 13, Tables 3-6).

### On the species of the genus *Heterorhabditis*

Considering the results of this study and the analyses of all the literature that describes new species of the genus *Heterorhabditis*, the updated list of the species of the genus, including their status, is as follows.

#### Type species of the genus

*Heterorhabditis bacteriophora* Poinar, 1976

= *H. argentinensis* Stock, 1993. Synonymized by Hominick (2002) based on molecular evidence provided by Adams et al. (1998). Synonymization status is supported by molecular data of Phan et al. (2003) and Achinelly et al. (2017).

#### Other species of the genus

*H. amazonensis* Andaló, Nguyen & Moino, 2006

*H. atacamensis* Edgington, Buddie, Moore, France, Merino & Hunt, 2011

*H. baujardi* Phan, Subbotin, Nguyen & Moens, 2003

= *H. somsookae* Maneesakorn, An, Grewal & Chandrapatya, 2015. Synonymized by Hunt and Nguyen (2016) based on the minor ITS sequence divergencies between *H. baujardi* and *H. somsookae.* Synonymisation status is further supported by the molecular data analyses carried out by Dhakal et al. (2020).

*H. beicherriana* Li, Liu, Nermut, Půža & Mráček, 2012

*H. egyptii* Abd-Elgawad & Ameen, 2005. This species was declared *species inquirenda* by Nguyen and Hunt (2007) but considered valid by Sudhaus (2011). As this species was described showing all diagnostic characters of adults and larvae stages, and it is morphologically distinct from all the other valid species, this species is also considered valid herein. The lack of molecular data, however, impairs its inclusion in future phylogenetic studies. Nevertheless, new species description should contrast morphological characters with this species.

*H. downesi* Stock, Griffin & Burnell, 2002

*H. floridensis* Nguyen, Gozel, Köppenhöfer & Adams, 2006

*H. georgiana* Nguyen, Shapiro-Ilan & Mbata, 2008

*H. hambletoni* (Pereira, 1937) Poinar, 1976

= *Rhabditis hambletoni* Pereira, 1937. This species was described showing all diagnostic characters of adults and larvae stages. It was transferred to the genus *Heterorhabditis* by Poinar (1976). As this species was described showing all diagnostic characters of adults and larvae stages, and it is morphologically distinct from all the other valid species, this species is considered valid herein. The lack of molecular data, however, impairs its inclusion in future phylogenetic studies. Nevertheless, new species description should contrast morphological characters with this species.

*H. indica* Poinar, Karunakar & David, 1992

= *Heterorhabditis brevicaudis* Liu, 1994. Several important diagnostic characters are missing and no molecular data are provided in the description of this species, although, it appears to be morphologically different from *H. downesi*, *H. baujardi*, and *H. mexicana* (Stock et al., 2002; Phan et al., 2003; Nguyen et al., 2004). Perhaps due to this reason, it was declared *species inquirenda* by Nguyen and Hunt (2007). A nematode population that shares several morphological characters with the original population used to describe the species was characterized more recently (Hsieh et al., 2009). ITS sequences are almost identical to the sequences of *H. indica*, justifying its synonymization (Hunt and Nguyen, 2016; Dhakal et al., 2020).

= *Heterorhabditis hawaiiensis* Gardner, Stock & Kaya, 1994. Not formally synonymized. However, synonymization status is supported by molecular data of Adams et al. (1998), Liu et al. (1999), and Phan et al. (2003), and multivariate analyses based on morphological characters of Stock and Kaya. (1996).

= *Heterorhabditis gerrardi* Plichta, Joyce, Clarke, Waterfield & Stock, 2009. Synonymized by Hunt and Nguyen (2016) based on the absence of ITS sequence divergencies. Synonymisation status is supported by further molecular data analyses carried out by Dhakal et al. (2020).

= *Heterorhabditis pakistanensis* Shahina, Tabassum, Salma, Mehreen & Knoetze, 2016. Synonymized by Hunt and Nguyen (2016) based on the minor ITS sequence divergencies between *Heterorhabditis pakistanensis* and *H. indica*. Synonymisation status is further supported by molecular data analyses carried out by Dhakal et al. (2020).

*H. marelatus* Liu and Berry, 1996

= *Heterorhabditis hepialius* Stock, Strong & Gardner, 1996. Synonymized by Stock (1997) based on morphological and morphometric anayses and cross-breeding tests. Synonymization status is further supported by molecular data of Adams et al. (1998) and Liu et al. (1999).

*H. megidis* Poinar, Jackson & Klein, 1987

*H. mexicana* Nguyen, Shapiro-Ilan, Stuart, McCoy, James & Adams, 2004

*H. ruandica* n. sp.

*H. noenieputensis* Malan, Knoetze & Tiedt, 2014

*H. safricana* Malan, Nguyen, De Waal & Tiedt, 2008

*H. taysearae* Shamseldean, Abou El-Sooud, Abd-Elgawad & Saleh, 1996

= *Heterorhabditis sonorensis* Stock, Rivera-Orduño & Flores-Lara, 2009. Synonymized by Hunt and Nguyen (2016) based on the minor ITS sequence divergencies between *H. taysearae* and *H. sonorensis*. Synonymisation status is further supported by molecular data analyses carried out by Dhakal et al. (2020).

*H. zacatecana* n. sp.

*H. zealandica* Poinar, 1990

= *Heterorhabditis heliothidis apud* Wouts, 1979 *nec* Khan, Brooks & Hirschmann, 1976. This species was reclassified as *H. zealandica* by Poinar (1990) as it is morphologically different from *Heterorhabditis heliothidis apud* (Khan et al., 1976).

#### Species inquirendae

*H. hoptha* (Turco, 1970) Poinar, 1979.

= *Neoaplectana hoptha* Turco, 1970

This species was poorly described. The original description lacks differentiated description of all diagnostic characters of adult and larval stages. According to this, this species should remain on the list of *species inquirendae*.

*H. poinari* Kakuliya and Mikaia, 1997. This species was poorly described. The original description lacks differentiated description of all diagnostic characters of adult and larval stages. According to this, this species should remain on the list of *species inquirendae*.

*H heliothidis* (Khan, Brooks & Hirschmann, 1976) Poinar, Thomas & Hess, 1977.

= *Chromonema heliothidis* (Khan, Brooks & Hirschmann, 1976)

This species was synonimized by Akhurst 1987 based on differential electrophoretic patterns of nematode lysates. However, the original description of *H. bacteriophora* carried out by Poinar (1976) shows males with very anterior GP1, while in its synonymized species *H. heliothidis* (Khan, Brooks & Hirschmann, 1976) Poinar, Thomas & Hess, 1977 the GP1 appears more posterior (Khan et al., 1976; Poinar, 1976). Probably both species are not conspecific. We therefore declare *H. heliothidis* (Khan, Brooks & Hirschmann, 1976) Poinar, Thomas & Hess, 1977 *species inquirenda.*

#### Nomina nuda

*H. downesi* Hass et al. 2001 *nec H. downesi* Stock, Griffin & Burnell, 2002

*H. minutus* Prabhuraj, Viraktamath & Kumar, 2002.

## Conclusions

The results of our study uncover the low levels of interspecific variation in some regions of the rRNA genes, especially in the D2–D3 expansion segments of the 28S rRNA, and also uncover the almost absent intraspecific variation of these sequences in the nematodes of the “*Bacteriophora*-group”. Mitochondrial genes such as *COI* provide better phylogenetic resolutive power, even at the population level, highlighting their great potential for the taxonomic characterization of closely related species of the genus *Heterorhabditis*. The threshold for species delimitation using *COI* sequences has been proposed to be around 94% (Pentinsaari et al., 2016). Using this threshold, we can clearly assign the Mexican and the Rwandan nematodes to new taxa within the “*Bacteriophora* group”. However, the sequence similarity scores of the Mexican and the Rwandan nematodes is between 97.6% and 98.2%. These scores are higher than the proposed 94% threshold but are consistent across nematode isolates and significantly lower than the intraspecific variations, prompting the question of whether the Rwandan and the Mexican nematodes should be classified into two different species, or into the same. Based on the results of the self-crossing and cross-hybridization experiments, and on the evident morphological and morphometric differences between these two groups of nematodes, we conclude that they indeed represent two distinct biological species. Thus, the boundary that delimits species in the genus *Heterorhabditis* is around 97% to 98% sequence similarity in the *COI* genomic sequence, and the Rwandan and the Mexican nematodes represent two new species, *Heterorhabditis ruandica* n. sp and *H. zacatecana* n. sp.

## Supplementary Material

Supplementary figures and tables can be retrieved from: https://doi.org/10.5281/zenodo.5614704

## Conflicts of interest

The authors declare no competing interests.
